# Emulsions stabilized by pea protein-rich ingredients as an alternative to dairy proteins for food sustainability: Unveiling the key role of pea endogenous lipids in the surface-induced crystallization of milk fat

**DOI:** 10.1016/j.crfs.2024.100921

**Published:** 2024-11-14

**Authors:** Christelle Lopez, Magalie Weber, Hanitra Rabesona, Javier Pérez, Franck Artzner, Thomas Bizien

**Affiliations:** a INRAE, BIA, F-44316, Nantes, France; b Synchrotron Soleil, L'Orme des Merisiers, Saint-Aubin BP48, F-91192, Gif-sur-Yvette, France; c IPR, UMR 6251, CNRS, University of Rennes 1, F-35042, Rennes, France

**Keywords:** Plant protein ingredient, Plant endogenous lipid, Emulsifier, Interface, Phospholipid, Solid fat phase, Lipid droplet, Crystallization in emulsion

## Abstract

In the current context of food transition, the growing demand of consumers for sustainable plant-based protein sources has stimulated interest of food scientists in plant protein ingredients as alternatives to dairy protein ingredients. In this study, we hypothesized that the crystallization properties of dairy emulsions could be affected by the chemical complexity of commercially available pea protein-rich ingredients that contain proteins but also endogenous lipids. Dairy emulsions (30 %wt milk fat) stabilized either by a pea protein isolate or dairy proteins were prepared, their microstructure and interfacial composition were characterized. The crystallization and melting properties of milk fat in anhydrous state and in the emulsions were examined by the combination of differential scanning calorimetry (DSC) and synchrotron-radiation X-ray diffraction as a function of temperature (SR-XRDT). The results revealed differences in the milk fat crystallization properties in emulsion as a function of the ingredient used and highlighted a specific role played by pea endogenous lipids. The pea protein-rich ingredient contained 12.1 %wt endogenous lipids including 56.2 %wt polar lipids, 40.7 %wt triacylglycerols (TAGs) and 3.1 %wt plant sterols. The partitioning of pea endogenous lipids occurred upon emulsion formation as a function of their polarity: liquid unsaturated fatty acid rich pea TAGs mixed with milk TAGs in the core of the lipid droplets while pea polar lipids migrated at the TAGs/water interface together with pea proteins. Pea polar lipids were composed of saturated high melting temperature (T_m_) and unsaturated low T_m_ molecular species. High T_m_ pea polar lipids exhibited a phase transition on cooling (from L_α_/expansed to L_β_/condensed) and acted as interfacial templates for surface heterogeneous nucleation and crystal growth of high crystallization temperature milk TAGs. The key interfacial and functional roles played by pea endogenous lipids present in the protein isolate were demonstrated. This study highlights the importance to examine the chemical composition and the properties of plant-based ingredients that are increasingly used for sustainable food formulations.

## Highlights

• Plant-based protein ingredients are used as alternatives to milk proteins for sustainable food emulsions.
• The crystallization properties of milk fat in emulsion are affected by pea endogenous lipids.
• Pea protein isolates contain 12 %wt endogenous lipids that partition upon emulsion preparation.
• The polar lipid fraction of pea endogenous lipids is involved in emulsion surface properties.
• High melting temperature polar lipids act as templates for heterogeneous crystallization in emulsion.

## Graphical abstract

**Figure:**
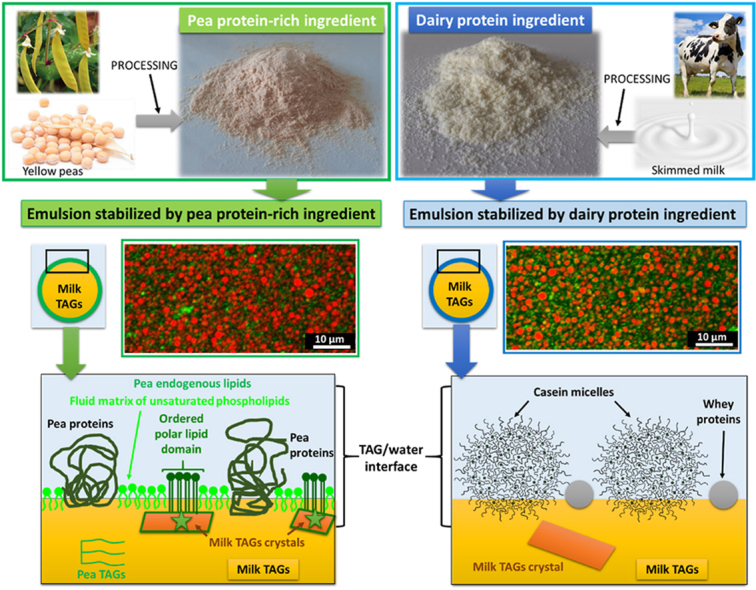

## 1 Introduction

There is growing societal consciousness about the impacts of the agricultural and food manufacturer practices on the carbon footprint. This leads to the food transitions, including the protein transition from animal towards plant-based proteins. Growing trends in the food sector area have emerged in recent years towards more sustainable food systems integrating higher amounts of plant-based food products in the diet for human nutrition. Also, concerns about animal welfare are leading to increasing efforts to reduce the part of animal proteins and animal derived foods in the diet (i.e., meat, milk, dairy products and egg products) (Hölker et al., 2019). Environmental sustainability, threat of climate change, global health while ensuring food security for the world's growing population, and ethical considerations are among the most important driving forces behind this dietary shift (Springmann et al., 2018). The demand for plant protein sources, including pulses which are among the most protein-rich plants, that could substitute animal proteins from milk, meat and eggs has been increasing (Boye et al., 2010; Vogelsang-O’Dwyer et al., 2021). Furthermore, pulse consumption is strongly encouraged by the Food and Agriculture Organization due to adequate nutritional composition, relatively low prices, and benefits for maintaining soil health (Calles et al., 2019). The current context of food transition promotes the use of plant protein ingredients for the development of plant-based foods as alternatives to dairy products (McClements et al., 2019). For the specific case of dairy products, allergies to milk proteins as well as lactose intolerance have led consumers to search for healthier alternatives. The formulations of mixed plant and dairy foods, composed of mixtures of plant and dairy proteins with lipids of plant (vegetable oils) or dairy (milk fat) origins for food colloid design, are also emerging (Le Roux et al., 2020; Hinderink et al., 2021; Canon et al., 2022; Grasberger et al., 2022). A recent study reported the life cycle assessment of new fermented food products mixing cow milk and pea protein sources to examine the environmental interest to use plant-based ingredients in dairy formulations (Huguet et al., 2023). It is therefore of interest to address research questions about the impact of substituing dairy proteins by plant-based proteins on the oil-in-water (O/W) emulsion physico-chemical and functional properties, including the crystallization properties of dietary lipids in emulsions.

Many foods are O/W emulsions, e.g. cream, cream desserts, yogurts, spreads, infant formula and mayonnaise, in which dietary lipids are mostly the triacylglycerols (TAGs; esters of glycerol and fatty acids). In O/W emulsions, the TAG phase is dispersed in the form of individual droplets surrounded by an aqueous phase. The interface between the TAG phase entrapped in the droplets and the surrounding aqueous phase plays a key role in the physico-chemical stability of the emulsions. In food emulsions, the interfacial layer is generally composed of dietary proteins and other emulsifiers (e.g., polar lipids also called lecithin, mono and diacylglycerols). The amphiphilic nature of the proteins allows them to be used as natural emulsifiers to help reduce interfacial tension and stabilize the O/W interface. Exploring the emulsifying properties of pulse protein-rich ingredients is therefore of interest for the developpement of new plant-based foods (Burger and Zhang, 2019).

The TAG phase of food products is often partially crystallized at the storage and consumption temperatures, as it is the case for chocolate (cocoa butter), margarine (mixture of partially hydrogenated vegetable oils) and dairy products (milk fat). The TAG crystals are involved in the structure, the texture and the organoleptic properties of the food products. The crystallization of milk fat contributes to the texture of dairy products by providing firmness as compared to liquid vegetable oils, for example in yogurt (Barrantes et al., 1996; Canon et al., 2022). Such TAG crystallization plays an important role in the manufacture, storage, transport and consumption of emulsified food products, as well as in their physical stability. It is therefore of great interest to identify and understand the mechanisms that impact the crystallization behaviour of TAGs in dispersed systems such as O/W emulsions (Douaire et al., 2014). In food emulsions including dairy emulsions, parameters such as the size of the lipid droplets (Lopez et al., 2002), the presence of molecules at the TAG/water interface (Lopez and Ollivon, 2009a), and the kinetics of cooling (Lopez et al., 2002) have been reported to affect the crystallization properties of TAGs in O/W emulsions (Lopez, 2020a).

Protein-rich ingredients prepared from pulses are becoming the most appropriate alternative to protein ingredients of animal origin. This is due to their high nutritive quality, good techno-functional properties (including emulsifying, foaming and gelling properties), low cost and high commercial availability. Pulse protein ingredients, for example from pea and other pulses (faba bean, lentil, lupin, chickpea and common bean), have received much interest for their functionality and their potential as nutritious ingredients by providing a source of essential amino acids and bioactive peptides (Vogelsang-O’Dwyer et al., 2021). Plant-based protein ingredients with high contents in proteins, such as concentrates (protein content 49–70% of dry matter) and isolates (protein content above 70% of dry matter), are increasingly being recognized for their potential as functional ingredients and to replace animal proteins. The use of pulse protein concentrates or isolates opens up the possibility of formulating foods to a target nutritional composition. However, for specific food applications (e.g. infant milk formula) the presence of starch and fibers in the plant protein concentrates is not suitable and the isolates of plant proteins are therefore preferred to reach the functional (physical stability of the O/W emulsion, viscosity) and nutritional objectives (Le Roux et al., 2020).

At present, the only widely available pulse protein concentrates or isolates prepared at the industrial scale for commercial needs are derived from pea. Peas (*Pisum sativum* L.) are one of the most widely cultivated and consumed legumes in the world, which are grown and consumed mainly in Canada, France, China, Russia, and the United States (Burger and Zhang, 2019). Pea seeds meet sustainability demand, the pea protein-rich ingredients display promising functional properties, and pea proteins have the advantage of being non-allergenic wich boost their use as ingredient for food applications (Shanthakumar et al., 2022). The processing of pea seeds into pea protein isolates governs the final composition of the ingredients, including the amount of non-protein components (fibers, starch, lipids, polyphenols), and their functional properties. The enrichment in pea endogenous lipids in the protein isolates which results from processing has been reported 40 years ago (Coxon and Wright, 1985; Hoover et al., 1988; Keuleyan et al., 2023) and is known by the manufacturers (patent FR3104906A1, ROQUETTE FRERES, 2019, 3). The presence of pea endogenous lipids in protein isolates could have physico-chemical, sensory and functional consequences. The high oxidation potential of these endogenous lipids that contain polyunsaturated fatty acids (Hoover et al., 1988), could notably lead to rancidity via autoxidation and lipoxygenase activity, and to the production of bitter off-flavor that raises concern for the utilization of pea protein isolates in foods. Moreover, due to the complexity of their composition, and the presence of many surface-active components (i.e., polar lipids), the pea endogenous lipids could play a key role at the surface of lipid droplets in food emulsions, including in the crystallization properties of TAGs.

In this work dedicated to the development of O/W emulsions stabilized by plant-based ingredients as alternatives to dairy proteins, we hypothesized that constituents present in commercially available pea protein-rich ingredients could affect the crystallization properties of milk TAGs dispersed in lipid droplets. The objectives of this study were therefore: (i) to formulate O/W emulsions stabilized by either dairy or pea protein-rich ingredients and characterized by similar droplet size distributions, (ii) to conduct a comparative analysis of the TAG crystallization and melting characteristics in the emulsions stabilized by either dairy or pea protein ingredients, and (iii) to elucidate the role played by the pea endogenous lipids on the crystallization properties of milk TAGs. Our strategy consisted in using biophysical techniques including differential scanning calorimetry (DSC) and X-ray diffraction (XRD). These techniques have been demonstrated over the years to be particularly suitable to provide an insight into the thermodynamics of fat phase transitions and to identify TAG crystals in bulk and in emulsion (ten Grotenhuis et al., 1999; Lopez et al., 2001; Douaire et al., 2014; Lopez, 2020a). Moreover, DSC and synchrotron radiation XRD recorded as a function of temperature have been previously reported to be highly sensitive techniques for the detection of surface-induced crystallization of TAGs dispersed within emulsion lipid droplets (Lopez and Ollivon, 2009a; Douaire et al., 2014).

## 2 Materials and methods

### 2.1 Materials

The milk fat used in this study was a natural blend of triacylglycerols (TAGs: 98% of milk fat) corresponding to a high melting point fraction of milk fat that was provided by Corman S.A. (Limbourg, Belgium).

The dairy and pea protein ingredients were commercially available ingredients provided in the form of powders and used as received.

The dairy protein ingredient corresponded to an industrial food grade skimmed milk powder and was kindly provided by Lactalis (Lactalis Ingredients, Retiers, France). According to the manufacturer, the powder contained 37.3 %wt dairy proteins (N x 6.25), 53.9 %wt lactose, 8.3 %wt ash, and 0.5 %wt lipids in the dry basis.

The pea (*Pisum sativum* L.) protein-rich ingredient Nutralys® S85 Plus N was an isolate of pea proteins manufactured using wet fractionation and purchased from Roquette (Roquette Frères S.A., Lestrem, France). The ingredient Nutralys® S85 Plus N was chosen since it is the most soluble pea protein-rich ingredient at a large range of pH values for nutritional applications with a protein content above 3 %wt. According to the manufacturer, the pea protein isolate Nutralys® S85 Plus N contained 84 %wt proteins (N x 6.25), 10 %wt lipids, and 1 %wt fibers in the dry basis, with an absence of starch.

Sodium azide (0.02 %wt) was added to the aqueous suspensions of the ingredients in order to prevent bacterial growth during storage.

### 2.2 Preparation of the O/W emulsions

Oil-in-water (O/W) emulsions were prepared as follows. The dairy protein ingredient and the pea protein-rich ingredient were dispersed in ultra pure water by weighting the amount of powder necessary to reach 4 %wt proteins in the aqueous suspensions (this protein content is similar to bovine mik (Lopez et al., 2014)), and permitted the preparation of the emulsions containing 30 %wt fat). The protein aqueous suspensions have been left under steering with a magnetic stirrer for 24 h. Milk fat has been heated at 70 °C for 1 h in order to ensure complete melting of the TAGs. O/W emulsions were prepared by mixing the melted TAGs and the protein aqueous suspensions to reach 30 %wt fat (e.g., to prepare 100 g of final emulsion, 30 g of fat were weighted, then 70 g of the aqueous suspension of the protein ingredient were added to reach 100 g). Experimental reasons governed the fat concentration of 30 %wt since studying the crystallization properties of TAGs in emulsion by DSC and XRDT techniques is challenging and requires a good signal/noise ratio (Lopez, 2020a). The O/W emulsions were prepared using a multi-step process. First, pre-emulsification was performed by high-shear mixing using the rotor stator system Polytron PT 3000 (Kinematica, Littau, Switzerland) equipped with a 12 mm head working at 25,000 rpm for 30 s at 50 °C. Then, a two-stage laboratory high-pressure homogenizer (PandaPlus 1000, GEA Niro Soavi, Italy) was used. Homogenization was performed at 50 °C at pressures varying from 3.5 to 5 MPa in order to have a similar emulsion lipid droplet size distribution for all the interface compositions (i.e., dairy protein ingredient, pea protein-rich ingredient, dairy protein ingredient + pea endogenous lipids).

In addition, O/W emulsions were specifically prepared to examine the impact of the pea protein-rich ingredient on the mechanisms of milk TAGs crystallization and melting.

O/W emulsions were prepared to investigate the impact of (i) the whole pea endogenous lipids (noted pea-EL) contained in the pea protein-rich ingredient (protocol used for their recovery detailed section 2.8.2.), and of (ii) the polar lipid fraction of pea-EL (protocol used for their recovery detailed section 2.8.2.). The whole pea-EL or the polar lipid fraction of pea-EL were added to the dairy protein ingredient aqueous suspension at a similar amount than in the aqueous suspension of the pea protein-rich ingredient. The emulsions were prepared by using the same protocol as described above.
O/W emulsions were also prepared by varying the amount of the pea protein-rich ingredient to modulate the amounts of pea proteins (2, 3 or 4 %wt in the aqueous suspension) and pea-EL (0.7, 1 or 1.3 %wt of total lipids) in the systems. The emulsions were prepared by using the same protocol as described above.

### 2.3 Confocal laser scanning microscopy (CLSM)

The microstructures of the aqueous suspensions of the pea protein-rich ingredient and dairy protein ingredient and of the respective O/W emulsions were examined by confocal laser scanning microscopy (CLSM) using a Nikon microscope (Nikon A1R; Nikon, Champigny sur Marne, France). The X20 air, X40 water and X60 oil immersion objectives were used. The CLSM images were acquired and processed with the NIS-Elements viewer & imaging software version 5.20.01 (Nikon Instruments Inc.). The fluorescent dye Nile Red (5H-benzo-alpha-phenoxazine-5-one,9-diethylamino; 100 μg/mL in propanediol; Sigma Aldrich, St Louis, MO, USA; excitation wavelength = 560 nm) was used to stain the lipids in the aqueous suspensions of the ingredients and the TAGs dispersed within lipid droplets in the O/W emulsions. The fluorescent dye Fast Green FCF (10 mg/mL in water; Sigma-Aldrich, St. Louis, MO, USA; excitation wavelength = 636 nm) was used to stain the proteins in the aqueous suspensions of the pea and dairy protein ingredients and in the O/W emulsions. The samples were kept at room temperature for 30 min prior to microscopic observations.

### 2.4 Particle size measurements

The size distributions of the emulsion lipid droplets were determined with a laser diffraction analyser (Horiba LA-960V2, Retsch Technology, Haan, Germany). The refractive indices used were 1.458 for the milk fat and 1.33 for the continuous phase (water). The samples were characterized in two conditions: (i) in water and (ii) after 10-fold dilution in sodium dodecyl sulphate (SDS 1 %wt) to avoid flocculation between the lipid droplets and to determine the size distribution of individual lipid droplets. The samples were added to ultrapure water for the particle size measurements. The measurements of each sample were performed in triplicate at room temperature to calculate the mean diameters.

### 2.5 Zeta potential

For zeta-potential (ζ-potential) experiments, the emulsions were diluted in ultra pure water (100 μL emulsion in 50 g water). About 1 mL of each sample was filled into a cuvette. The cuvette was placed into the chamber of a Zetasizer Nano ZS (Malvern, Germany), and equilibrated at 20 °C for 5 min before measurements. The zeta potential was calculated from the electrophoretic mobility of the lipid droplets according to the Smoluchowski approximation and Henry's law. The measurements were run five times at 20 °C.

### 2.6 Thermal analysis: differential scanning calorimetry (DSC)

A DSC Q100 apparatus (TA Instruments, Newcastle, DE) was used to perform DSC measurements on anhydrous milk fat, O/W emulsions, pea endogenous lipids (noted pea-EL), mixtures of anhydrous milk fat and pea-EL, and vesicles of polar lipids fractionated from pea-EL. For all the DSC measurements, an empty and hermetically sealed aluminum pan was used as reference. The DSC Q100 calorimeter was calibrated with indium (ΔH = 28.41J/g; Melting point = 156.66 °C). Anhydrous milk fat was heated at 65 °C to ensure complete melting of the TAGs and 25–35 mg of the melted fat were loaded into aluminum pans (TA Instruments) that were hermetically sealed. The emulsions (about 25–35 mg accurately weighted) were loaded into aluminum pans that were hermetically sealed. Anhydrous pea-EL as well as the mixtures of milk fat and pea-EL (1.3 %wt and 3 %wt) were heated at 65 °C, then 15–20 mg were loaded into aluminum pans that were hermetically sealed. The DSC pans were introduced in the calorimeter at 20 °C. The protocol used to characterize the thermotropic behaviors of the anhydrous lipids and O/W emulsions was as follows: i) heating from 20 °C to 65 °C at 10 °C/min, ii) isotherm at 65 °C for 5 min, iii) cooling from 65 °C to −5 °C at 3 °C/min, iv) heating from −5 °C to 60 °C at 3 °C/min. Data analysis, e.g. determination of the temperatures of the peaks, was performed using TA Universal Analysis sofware. The DSC experiments were performed at least in triplicate for each sample, by preparing and analysing at least three independent pans.

### 2.7 Structural analysis: temperature-controlled synchrotron-radiation X-ray diffraction (XRD)

X-ray diffraction (XRD) experiments were performed with the high brilliance of the SWING beamline at the Soleil synchrotron facility (proposal 20220764, 16–17 sept 2022). A EIGERX 4M detector was set at 0.528 m from the sample and the monochromator was set at 16 KeV (David and Pérez, 2009). A HFSX350-CAP Linkam oven (Linkam Scientific Instruments Ltd, Waterfield, UK) was used to perform synchrotron-radiation XRD measurements as a function of temperature (XRDT) that is necessary to examine the crystallization and melting properties of TAGs. The samples of anhydrous milk fat, O/W emulsions and vesicles of polar lipids fractionated from the pea endogenous lipids were loaded in thin quartz capillaries of 1.5 mm diameter (GLAS W. Muller, Berlin, Germany). The capillaries were inserted into the Linkam oven for the XRD experiments. The samples were heated to 65 °C, cooled from 65 °C to −5 °C at 3 °C/min (375 patterns, 1 pattern each 0.2 °C, 2 s exposure time and 2 s gap time) and then heated at 3 °C/min from −5 to 65 °C (375 patterns, 1 pattern each 0.2 °C, 2 s exposure time and 2 s gap time). For the vesicles of pea polar lipids, XRD experiments were performed at 50 °C and 20 °C (5 patterns, 2 s exposure time and 2 s gap time). Diffraction patterns were recorded for reciprocal spacing q varying between 0.023 and 2.4 Å^−1^, that is, repetitive distances d = 2 π/q ranging from 136 to 1.3 Å. This scattering vector q range covered both the small and wide-angles regions of interest to characterize the crystalline structures of milk TAGs or the lamellar structures of polar lipids, and to identify the packing of the acyl chains, respectively. 1D XRD curves were obtained by circular averaging of the 2D images using the Foxtrot software. R software (R Foundation for Statistical Computing, Vienna, Austria) was used to display the three-dimensional plots of XRD patterns as a function of temperature. The positions and the middle height peak width (MHPW) of the Bragg reflections were determined with PeakFit software (Jandel Scientific, Germany).

### 2.8 Chemical analysis

#### 2.8.1 Interfacial protein profiles

##### 2.8.1.1 Preparation of the protein-coated lipid droplets before gel electrophoresis

For the determination of the interfacial protein composition of the lipid droplets (prepared with the pea protein-rich or the dairy protein ingredients), unadsorbed proteins were removed by washing according to a method adapted from Patton and Huston (1986) and previously described (Lopez et al., 2021). Briefly, 10 g of the emulsions were mixed with 10 g of a solution containing containing 50 %wt of sucrose. Then, in 50 mL plastic centrifuge tubes, 15 g of the treated emulsion were delivered under 25 g of a solution containing 5 %wt of sucrose. The tubes were centrifuged at 2500 *g* for 20 min at 25 °C to recover washed lipid droplets at the top of the tubes.

##### 2.8.1.2 Gel electrophoresis

The interfacial protein profiles of the lipid droplets previously washed as described in the previous paragraph, and the protein profiles of the dairy and pea ingredients aqueous dispersions used to prepare the O/W emulsions were determined by SDS-PAGE with gels gradient 4–15% Mini-Protean TGX Precast using Mini-Protean Tetra Cell sys-tem (Bio-Rad Life Science, France). All reagents were from Bio-Rad Life Science. The samples were diluted with 2x Laemmli sample buffer in denaturing conditions and both in reducing conditions with 2-mercaptoethanol 5 % (a reducing agent that breaks down the disulfide bonds), and in non-reducing conditions in absence of 2-mercaptoethanol. The running buffer contained 25 mM Tris, 192 mM glycine, 0.1 % SDS according to (Laemmli, 1970) protocol. The samples were boiled for 5 min at 100 °C to ensure total denaturation of the proteins, and then the samples were loaded onto the precast 4–15% gradient gel. Molecular weight (MW) protein markers from MW 10–250 kDa (Precision Plus Protein Standards, All Blue, Bio-Rad Life Science, France) and from MW 14.4–116 kDa (unstained Molecular weight marker, Euromedex, Souffelweyersheim, France) were used for MW calibration. The migration was carried out under standard conditions at 200 V for 45 min. The gel was stained with Coomassie Brilliant Blue G-250 staining solution for 2 h with gentle agitation on platform rocker according to (Lawrence and Besir, 2009). Then each gel was rinsed with distilled water before scanning on a flat-bed scanner (Image Scanner iii, GE Healthcare Europe, Velizy-Villacoublay, France).

#### 2.8.2 Lipid analysis

##### 2.8.2.1 Extraction of total pea endogenous lipids and fractionation

###### 2.8.2.1.1 Extraction of total lipids

Total lipids present in the pea protein isolate Nutralys® S85 Plus N ingredient, that were considered as whole pea endogenous lipids (noted pea-EL), were extracted in the laboratory in order to perform additional experiments: (i) to decipher their composition, (ii) to characterize their thermal and structural properties by DSC and XRD, and (iii) to determine their impact on the crystallization properties of milk TAGs in anhydrous state and in O/W emulsions. The pea-EL were recovered by solvent extraction using a multi-step protocol adapted from Folch et al. (1957). The Nutralys® S85 Plus N ingredient was hydrated at 20 %wt in ultrapure water under magnetic stirring for 12 h. The ingredient aqueous suspension has been homogenized at 10 MPa (PandaPlus 1000, GEA Niro Soavi, Italy) for 5 min recirculation (average of 10 cycles), to improve its dispersibility (i.e., break down of large poorly dispersed powder grains) before lipid extraction. The chloroform/methanol (2:1, v/v) solvent was added to the homogenized aqueous suspension, the mixture was stirred for 1 h and filtrated under vacuum to retrieve the permeate liquid phase. The retentate of filtration was recovered and the procedure was repeated three times to ensure the recovering of total pea-EL. The permeates were transferred into funels where a solution of NaCl (0.73 %wt) was added, and let to decant during 36 h at 4 °C. The bottom chloroform phase containing the pea endogenous lipids was collected and the solvents were evaporated under vacuum in a water bath at 40 °C (R-100, Rotavapor, Büchi, France), and then under nitrogen (N-Evap 111, Organomation, USA). The lipid extract corresponding to pea-EL was weighted to determine the total lipid content in the pea protein-rich ingredient Nutralys® S85 Plus N. This protocol of lipid extraction was repeated three times.

###### 2.8.2.1.2 Fractionation of lipid classes as a function of their polarity

The total lipid extracts corresponding to pea-EL (n = 3) were brought to silica gel column chromatography using Sep-Pak silica cartridge (1 g, Waters Corp. Milford, MA, USA), using a protocole adapted from Juaneda and Rocquelin (1985). An amount of 100 mg of total lipids was used. Chloroform was used to recover the neutral lipids that corresponded to triacylglycerols (TAGs), then methanol was used to collect the polar lipids, i.e., the phospholipids and glycolipids. Each fraction was dried under vacuum.

##### 2.8.2.2 Fatty acid profiles

The fatty acid profiles of milk fat, whole pea-EL, pea-EL TAGs and polar lipids were determined by a gas chromatography (GC) procedure after methylation with cold methanolic solution of potassium hydroxide. The fatty acid methyl esters (FAMEs) were analyzed by GC (Focus GC, Thermo Electron Corporation, Massachusetts, USA) equipped with a split injector (ratio of 1/20), a CPCil 88 Varian capillary column (50 m × 0.25 mm with a 0.2-μm thick film; Chrompack, Middelburg, The Netherlands) and 1 mL/min of helium as carrier gas. FAME were analyzed using a flame ionization detector and ChromCard Data System (Thermo Electron Corporation, Massachusetts, USA). The FAME were identified using a mixture of methyl esters as external standard (Mixture ME 100, Larodan, Sweden). The GC analysis was performed in triplicate for each extract of total lipids.

##### 2.8.2.3 TAG molecular species

The analysis of the molecular species of TAGs in milk fat and pea-EL was conducted by the official assay described for the separation, identification, and quantitative determination of individual TAGs in edible fats and oils using HPLC (AOCS Ce5c-93, 2022), as previously detailed (Lopez et al., 2023). An HPLC-ELSD (Agilent 1200, Agilent Technologies, Santa Clara, CA, USA) equipped with a C18 column (150 mm × 4.6 mm, 5 μm, Varian, Palo Alto, CA, USA) was used. The TAG extracts were prepared in hexane at 2 mg/mL, and then analyzed. The mobile phase consisted of acetonitrile and isopropanol (flow rate 0.8 mL/min). The initial mobile phase was held for 40 min at 60 % acetonitrile, changed linearly (40–80 min) to 55 %, returned to 60 % (80–85 min), and maintained for 5 min (85–90 min) at 60 %. The ELSD temperature was set at 55 °C (gain value of 8; gas flow rate 1.5 mL/min). TAGs were separated based on the equivalent carbon number (ECN), which is calculated with the formula: ECN = TCN − (2 DB), where TCN is the total carbon number and DB is the number of double bonds in the FA in each TAG. TAGs were identified by comparing their retention times with standard reference compounds. Relative content was reported as a percentage. From the lipid extracts, the molecular species of TAGs were determined in duplicate (2 injections in the HPLC); the results presented correspond to the mean of n = 6 chromatograms for pea-EL and milk fat.

##### 2.8.2.4 Polar lipid analysis

The quantification of total polar lipids and the determination of the individual polar lipid classes in the samples were performed using HPLC combined with an evaporative light scattering detector as previously described (Lopez et al., 2021). The identification of the phospholipids and glycolipids was carried out by a comparison with the retention time of pure standards. Quantitatification was performed using calibration curves.

##### 2.8.2.5 Plant sterols

The identification and quantification of total plant sterols were performed using gas chromatography with flame ionization (GC-FID) according to (ISO-122228-1, 2014, 12228), as previously described (Lopez et al., 2021). The different plant sterols were identified by comparing the retention times with the individual standards (Sigma-Aldrich). The internal standard 5α-cholestan-3β-ol (Sigma D6128) was used for quantification. From pea-EL lipid extracts (n = 3), the analysis of sterols was performed in triplicate. The results presented correspond to the mean of n = 9 values.

## 3 Results and discussion

### 3.1 From protein-rich ingredients to O/W emulsions: microstructure and interfacial protein composition

**Fig. 1:**
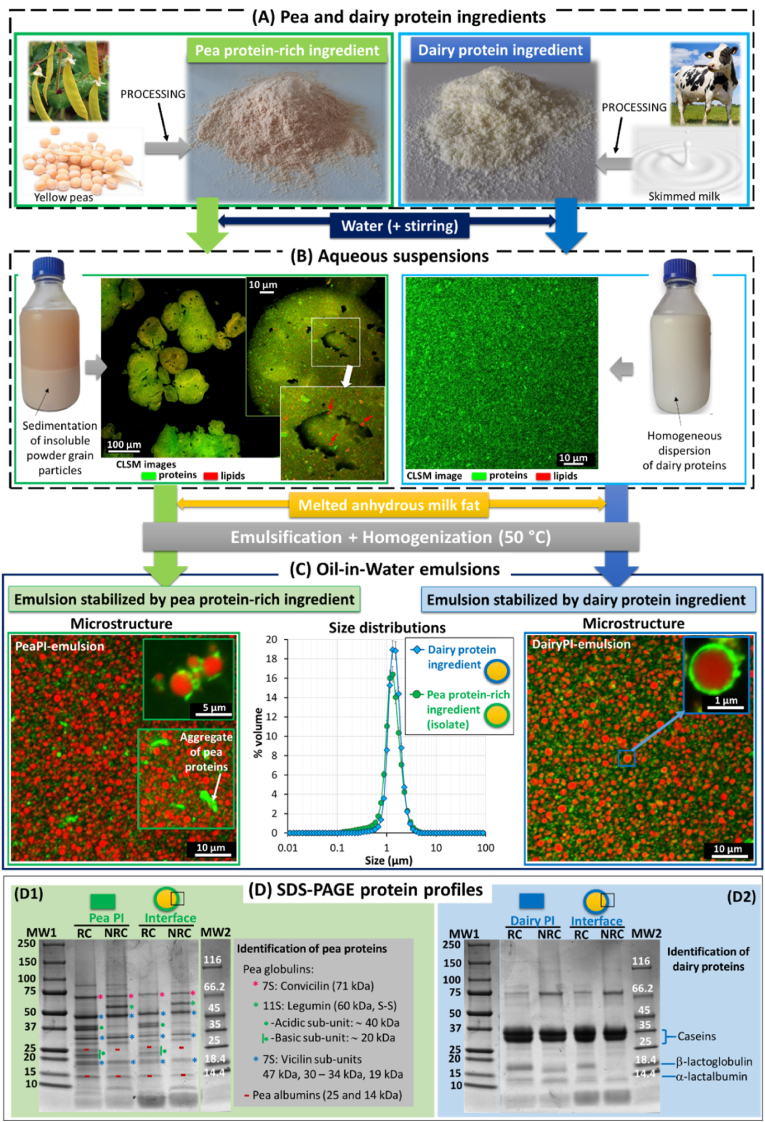
Preparation and characterization of the oil-in-water (O/W) emulsions. **(A)** Pea and dairy protein ingredients in the form of powders. **(B)** Aqueous suspensions of the pea and dairy protein ingredients. Pictures of the bottles containing the aqueous suspensions and confocal laser scanning microscopy (CLSM) images of the aqueous suspensions obtained with the fluorescent labelling of proteins with Fast green FCF (green colour) and of lipids with Nile red (red colour; highlighted by arrows). **(C)** Microstructure of the O/W emulsions: CLSM images of the O/W emulsions stabilized by the pea protein isolate or dairy proteins, the triacylcylcerols in the core of the lipid droplets are in red (Nile red fluorescent dye), the proteins are in green (Fast green FCF fluorescent dye); (Middle) Size distributions of the lipid droplets determined by laser light scattering. **(D)** SDS-PAGE protein profiles of the protein suspensions and interfacial protein profiles of the lipid droplets, (D1) prepared with the pea protein-rich ingredient and (D2) prepared with the dairy protein ingredient. Abbreviations: MW = molecular weight; NR = non-reducing conditions; RC = reducing conditions; Pea PI = aqueous suspension of the pea protein-rich ingredient; interface = Interfacial proteins adsorbed at the surface of the lipid droplets stabilized by the pea protein isolate (left) or the dairy protein ingredient (right); Dairy PI = Dairy protein ingredient. (For interpretation of the references to colour in this figure legend, the reader is referred to the Web version of this article.)

Fig. 1 presents the main steps involved in the preparation of the O/W emulsions from the pea protein-rich or the dairy protein ingredients, and characterizations. The microstructures of the aqueous suspensions of the ingredients and O/W emulsions as well as the interfacial protein profiles of the lipid droplets are also presented in Fig. 1.

The pea and dairy protein ingredients were dispersed in water to reach 4 %wt proteins. The pH values of the aqueous suspensions were pH 7.54 ± 0.11 for the pea protein-rich ingredient and pH 6.65 ± 0.05 for the dairy proteins. The aqueous suspension of the pea protein-rich ingredient exhibited phase separation with the sedimentation of insoluble powder grain particles observed at the macroscopic scale when the product was no longer left under stirring (Fig. 1B, left). CLSM images confirmed the presence of large powder grain particles poorly dispersed in water (Fig. 1B, left). CLSM images also showed pea endogenous lipids (pea-EL) entrapped in the protein matrix of the powder grain particles (Fig. 1B, left). The pea-EL assemblies observed at the microscopic scale could correspond to polar lipid coated TAGs droplets, vesicles and plant membranes. The large powder grain particles recovered in the suspension resulted from the low wettability of the pea protein isolate. The low dispersibility of the pea protein-rich ingredient was attributed to the protein isolate manufacturing process, mainly the isoelectric precipitation technique performed at acidic pH that leads to the formation of aggregates of pea proteins exposing their hydrophobic parts to the aqueous phase but could also be due to spray drying parameters (Boye et al., 2010). The dairy protein ingredient exhibited a high dispersibility in water as a result of the high wettability of skimmed milk powders and to the high solubility of dairy proteins (casein micelles and whey proteins) in water at pH 6.7. CLSM images showed a homogeneous distribution of the dairy proteins in the aqueous suspension (Fig. 1-B right).

Homogenization of the mixture of melted anhydrous milk fat with each aqueous suspension of protein ingredient induced the formation of O/W emulsions. The size distributions of the lipid droplets in the O/W emulsions stabilized by the pea protein-rich ingredient or dairy proteins were monomodal and centered on a similar mean diameter (Fig. 1 C, middle). In the emulsions stabilized by the pea protein-rich ingredient and the dairy proteins, the mean diameters were 1.29 ± 0.01 μm and 1.39 ± 0.01 μm, respectively. The zeta potential values were ζ = −35.9 ± 1.6 mV for the emulsions stabilized by components from the pea protein-rich ingredient and ζ = −19.6 ± 0.1 mV for the emulsions stabilized by the dairy proteins. These negative charges located at the surface of the lipid droplets contributed to strong electrostatic repulsions between the lipid droplets and favoured the physical stability of the O/W emulsions. CLSM images showed lipid droplets with a spherical shape, homogeneously distributed in the O/W emulsions and the presence of proteins both at the surface of the lipid droplets and in the surrounding aqueous phase (Fig. 1 C). Small protein aggregates were visible in the emulsions prepared with the pea protein-rich ingredient, showing that the large powder grain particles present in the aqueous suspension were disrupted by the high shear applied upon homogenization (Fig. 1 C), in agreement with the literature (Moll et al., 2021). CLSM images showed a heterogeneous repartition of pea proteins at the surface of the lipid droplets while the dairy proteins fully covered the surface of the lipid droplets (Fig. 1 C). In the emulsions prepared with either the pea protein-rich ingredient or the dairy proteins, the size of the lipid droplets did not evolve during their storage for 15 days (results not shown) and then did not show any coalescence. We deduced that the O/W emulsions were physically stable. This is in agreement with the good emulsifying properties of the pea protein-rich isolate already reported in the literature (Burger and Zhang, 2019).

The protein profiles of the aqueous suspensions prepared with the pea protein-rich ingredient and the dairy protein ingredient as well as the identification of the proteins adsorbed at the surface of the lipid droplets were determined by gel electrophoresis (SDS PAGE) in both non-reducing and reducing conditions (Fig. 1 D). The proteins were identified according to their molecular weight (MW) and the literature (Boye et al., 2010; Barac et al., 2015). In the aqueous suspension of the pea protein isolate (Fig. 1 D, left part), the proteins corresponded mainly to storage proteins that are named the globulins. The convicilin (7S) was present with MW 71 kDa. The legumin (11S) exhibited a unit at 60 kDa with a disulfide bond leading to acid subunits (40 kDa) and basic subunits (20 kDa) in reducing conditions. The vicilin (7S) exhibited several subunits with MW 47 kDa, 30–34 kDa, and 15–19 kDa. Water-soluble albumins (2S) have a low MW of 25 kDa, 14 kDa and 6 kDa (Boye et al., 2010; Barac et al., 2015). The comparative analysis of the SDS PAGE protein profiles of the pea proteins contained in the aqueous suspension of the ingredient and adsorbed at the surface of the lipid droplets did not reveal any difference in their composition and in the relative proportion of the different pea proteins (Fig. 1 D, left part). This indicated that both pea globulins and albumins were adsorbed at the TAG/water interface. These results are in agreement with previous works reporting the presence of vicilin, legumin and convicilin at the surface of emulsified lipid droplets (Chen et al., 2019). In the aqueous suspension of dairy proteins, the caseins (MW 25–35 kDa; 80 %wt of dairy proteins) and the main soluble proteins (20 %wt of dairy proteins) β-lactoglobulin (18.4 kDa) and α-lactalbumin (14 kDa) were identified (Fig. 1 D2), in agreement with the literature (Farrell et al., 2004). The caseins were the main proteins adsorbed at the TAG/water interface but the soluble proteins were also present.

### 3.2 Crystallization and melting properties of TAGs in anhydrous state and dispersed in lipid droplets stabilized by dairy or pea protein-rich ingredients

The impact of the interface, i.e. anhydrous state *versus* dispersion within individual lipid droplets and surface composition (dairy proteins or pea protein-rich ingredient), on the crystallization and melting properties of milk TAGs was examined by DSC combined with synchrotron-radiation XRD as a function of temperature (XRDT) to elucidate the thermal and structural properties, respectively.

#### 3.2.1 Crystallization behavior recorded on cooling from the melt

##### 3.2.1.1 Thermal properties

**Fig. 2:**
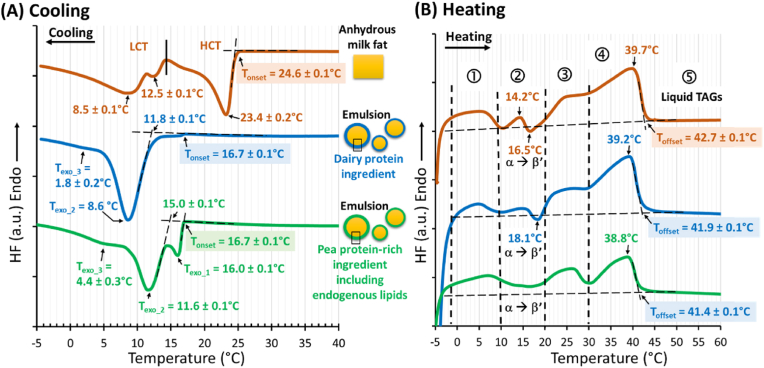
Differential scanning calorimetry (DSC) curves recorded **(A)** on cooling at 3 °C/min and **(B)** on subsequent heating at 3 °C/min of anhydrous milk fat (top), emulsion stabilized by dairy protein ingredient (middle) and emulsion stabilized by pea protein-rich ingredient (bottom). Abreviations: HCT = high crystallization temperature; LCT = low crystallization temperature; T_onset_ = initial temperature of crystallization; T_exo_: temperature at the minimum of the exotherm; T_offset_ = final temperature of melting; TAGs: triacylglycerols.

The DSC curves recorded on cooling at 3 °C/min of anhydrous milk fat and of the O/W emulsions stabilized either by the dairy proteins or the pea protein-rich ingredient are presented Fig. 2.

The DSC curve recorded on cooling of milk TAGs in their anhydrous state showed 2 main exothermic events (above and below 15 °C), with 4 successive exotherms. The crystallization of anhydrous TAGs was initiated at T_onset_ = 24.6 ± 0.1 °C with a minimum of the first exotherm at 23.4 ± 0.2 °C. The minimums of the other exotherms were recorded at 16.7 ± 0.1 °C, 12.7 ± 0.1 °C and 8.9 ± 0.3 °C. The two main exotherms were interpreted as the successive crystallizations of two groups of milk TAG molecules that segregated on cooling, i.e. the high crystallization temperature (HCT, above 15 °C) and the low crystallization temperature (LCT, below 15 °C) TAGs, in agreement with previous reports (ten Grotenhuis et al., 1999; Lopez et al., 2005, 2006; Lopez, 2020a).

On cooling of the O/W emulsions, the initial temperature of milk TAGs crystallization within the lipid droplets stabilized either by components from the pea protein-rich ingredient or dairy proteins was T_onset_ = 16.7 ± 0.1 °C. Compared to anhydrous milk TAGs, the DSC curves showed a delay in the induction of TAGs crystallization in emulsions (ΔT_onset_ = 24.6–16.7 °C = 7.9 °C), and exhibited different DSC profiles. The differences between the thermal behaviours of milk TAGs in anhydrous state and in O/W emulsions were interpreted as the impact of the dispersion of the TAGs within individual lipid droplets, as previously reported for milk TAGs (Lopez et al., 2002; Lopez, 2020a) and for a complex mixture of TAGs tailored to study the crystallization in O/W emulsions (Lopez et al., 2001). It has been previously observed and reported in the literature that oil contained within a droplet requires more supercooling than the equivalent bulk anhydrous fat, the most accepted explanation being that these droplets are statistically free of impurities, therefore preventing heterogeneous nucleation (Douaire et al., 2014). In O/W emulsions, cartalytic impurities can induce the formation of nucleation sites for crystallization within lipid droplets (Skoda and Van den Tempel, 1963; Sato, 2018). Interestingly, the DSC curves recorded on cooling of the emulsions stabilized by the dairy proteins or the pea protein-rich ingredient were different. The DSC curves recorded on cooling of the O/W emulsion stabilized by dairy proteins showed (i) a tiny exotherm with T_onset_ = 16.7 ± 0.1 °C, (ii) a main exotherm with T_onset_ = 11.8 ± 0. 1 °C and a minimum at T_exo_2_ = 8.6 ± 0.1 °C, and (iii) a residual exothermic signal was recorded below 5 °C, in agreement with literature (Lopez et al., 2002). For the O/W emulsion stabilized by the pea protein-rich ingredient, 3 successive exotherms were recorded on cooling. The first sharp exotherm started at T_onset_ = 16.7 ± 0.1 °C with a minimum at 16.0 ± 0.1 °C, the second exotherm started at T = 15.0 ± 0.1 °C with a minimum at T_exo_2_ = 11.6 ± 0.1 °C and the third exotherm exhibited a minimum at 4.4 ± 0.3 °C. In the lipid droplets stabilized by the pea protein-rich ingredient, the crystallization of TAGs was highlighted by a first exotherm and the other two exotherms recorded on cooling occurred at higher temperature than in the lipid droplets stabilized by the dairy proteins (ΔT_exo_2_ = 11.6–8.6 = 3.0 °C; ΔT_exo_3_ = 4.4–1.8 = 2.6 °C). Differences in the DSC curves recorded on cooling could not be attributed to differences in the size distributions of the lipid droplets since they were similar (Fig. 1 C). We therefore interpreted the first exotherm recorded on cooling of the O/W emulsion stabilized by the pea protein-rich ingredient (Fig. 2 A) as surface-induced heterogeneous nucleation and crystal growth, as previously reported in literature for other emulsion systems (Skoda and Van den Tempel, 1963; Lopez and Ollivon, 2009a; Douaire et al., 2014).

##### 3.2.1.2 Structural analysis: XRD

**Fig. 3:**
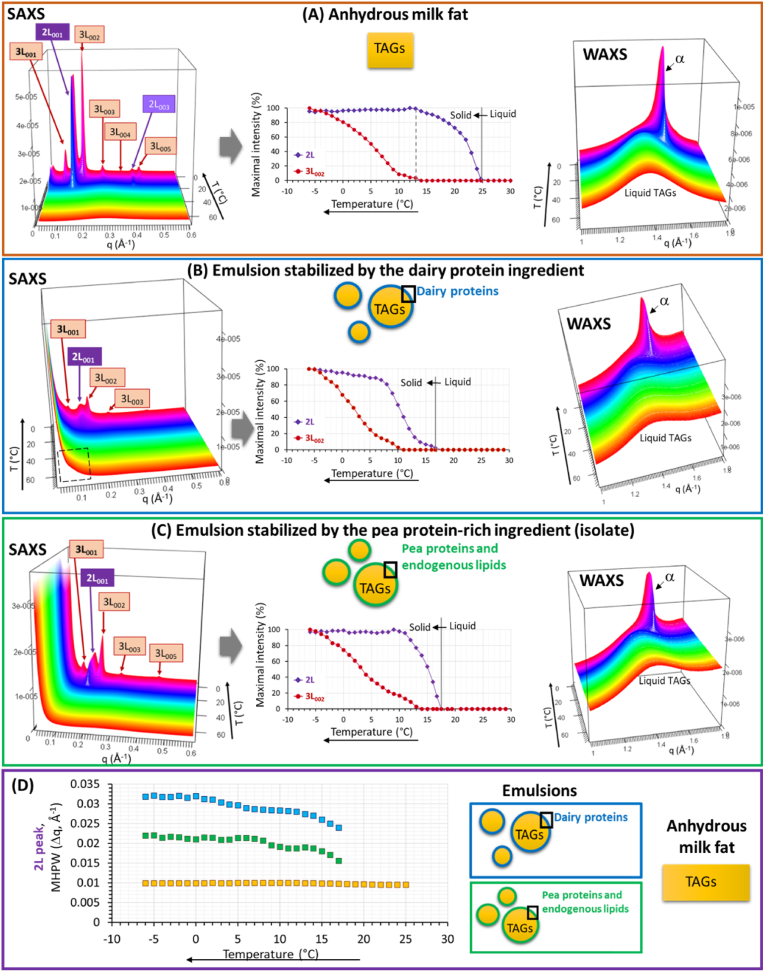
Three-dimensional plots of the synchrotron radiation X-ray diffraction (XRD) patterns recorded on cooling at 3 °C/min simultaneously at small (SAXS, left) and wide (WAXS, right) angles of **(A)** anhydrous milk fat, **(B)** emulsions stabilized by the dairy protein ingredient and **(C)** emulsion stabilized by the pea protein-rich ingredient; (middle) changes in the maximal intensity of the peaks corresponding to the first order of the 2L and 3L crystalline structures. The phases are identified in the figures and the reflexions are noted (Miller index; hkl). **(D)** Changes in the middle height peak width (MHPW expressed in Å^−1^) of the 2L peak as a function of temperature. Abreviations: TAGs = triacylglycerols; 2L = double chain length; 3L = triple chain length; T = temperature; q = scattering vector.

Fig. 3 shows the 3D-representations of the synchrotron-radiation XRD patterns recorded simultaneously at small and wide angles as a function of time on cooling between 65 and -5 °C at dT/dt = 3 °C/min of anhydrous milk TAGs and milk TAGs dispersed in the O/W emulsions stabilized either by dairy proteins or the pea protein-rich ingredient. The XRD patterns recorded at small and wide angles correspond to the longitudinal organization of TAG molecules and the lateral packing of fatty acids, respectively. The final temperature (e.g., −5 °C) was chosen to avoid ice formation in the O/W emulsions and to allow a comparison with the crystallization behaviour of milk TAGs in anhydrous state and dispersed in the emulsion lipid droplets. Fig. 3 also shows the evolution of the XRD peak parameters as a function of temperature, i.e. the maximal intensity of XRD peaks and the middle height peak width (MHPW).

###### 3.2.1.2.1 Crystallization of anhydrous milk TAGs

For temperatures above 25 °C, all the anhydrous milk TAGs were in their liquid state characterized by the recording at wide angles of a bump of X-ray scattering centered at 1.38 Å^−1^ and no diffraction peak at small angles. Below 25 °C, the successive formation and increase in intensity of diffraction lines corresponded to the progressive crystallization of TAGs in two different lamellar structures: α 2L and then α 3L, that were related to the main 2 exothermic events recorded by DSC (Fig. 2 A). The first lamellar structure formed on cooling was characterized at small angles by a peak located at 0.133–0.134 Å^−1^ (first order) and an other peak at 0.4 Å^−1^ (third order) that were attributed to a double chain length organization (2L) with a thickness of 47.2–46.8 Å and at wide angles by a single peak at 1.37 Å^−1^ (d = 4.19 Å) corresponding to an hexagonal chain packing (α polymorphic form). The evolution of the maximal intensity of this XRD peak showed an increase corresponding to a growth of α 2L crystals from 25 to 13 °C, associated to a decrease in the thickness of the bilayered crystals from 47.2 to 46.8 Å and then a stabilization (Fig. 3 A, middle). From 13 °C, the simultaneous appareance and increase in intensity of XRD peaks at small angles located at q = 0.0871 Å^−1^ (72.1 Å; 3L_001_), 0.1725 Å^−1^ (36.4 Å; 3L_002_), 0.2610 Å^−1^ (24.1 Å; 3L_003_), and 0.433 Å^−1^ (14.5 Å; 3L_005_), associated with the formation of a single peak at wide angles (q = 1.52 Å^−1^; d = 4.2 Å), were attributed to the formation of a triple-chain length organization with an hexagonal chain packing: α 3L (72.1 Å) with superior orders of Bragg reflections (noted 3L_hkl_). The increase in intensity of these XRD peaks was related to the growing of the α 3L (72.1 Å) crystals until the end of the cooling (Fig. 3 A, middle). The crystallization behaviour of milk TAGs in their anhydrous state characterized on cooling at 3 °C/min from the melt was in accordance with previous results examining the structural evolution of milk TAGs on cooling (Lopez et al., 2005, 2006). The first lamellar structure formed on cooling (α 2L) was attributed to the crystallization of HCT TAGs composed of trisaturated long chain fatty acids. The second types of crystals formed on cooling (α 3L) corresponded to LCT TAGs containing at least one unsaturated or short-chain fatty acid, as already described and discussed (Lopez et al., 2006).

###### 3.2.1.2.2 Crystallization of milk TAGs in emulsion lipid droplets stabilized by dairy proteins or by components from the pea protein-rich ingredient

The 3D views of the XRDT patterns recorded for the O/W emulsions clearly showed that the dispersion of TAG molecules within emulsion lipid droplets with 30 %wt fat induced a decrease in the XRD signal and an increase in the X-ray scattering at very small angles (q < 0.15 Å^−1^; highlighted in Fig. 3 B). This was attributed to scattering of the lipid droplets and of milk casein micelles present in the dairy protein ingredient (Fig. 3 B left). In the XRDT patterns, the absence of diffraction peaks for temperatures above 17 °C corresponded to TAG molecules in their liquid state within the emulsion lipid droplets. Whatever the composition of the interface, the simultaneous recordings from about 17 °C of a diffraction peak at about 46 Å (at small angles) and at 4.2 Å (at wide angles) corresponded to the crystallization of TAG molecules in the lipid droplets with the formation of a lamellar structure with a two-chain length organization (2L) of α form: α 2L (46 Å). In the lipid droplets stabilized by the pea protein-rich ingredient, the thickness of the 2L crystals decreased from 45.9 to 45.0 Å from its formation around 17 °C–7 °C, while it decreased from 45.9 to 43 Å in the lipid droplets stabilized by the dairy protein ingredient. From about 10 °C for the lipid droplets stabilized by the dairy proteins and from 13 °C for the lipid dropelts stabilized by the pea protein-rich ingredient, XRDT patterns recorded at small angles showed the increase in intensity of XRD peaks related by orders of diffraction corresponding the formation of α 3L (72.0–72.1 Å) structure. The analysis of the evolutions of the maximal intensity and MHPW of the XRD peak related to the first crystalline structure formed on cooling, i.e. the α 2L (46 Å), showed differences between the emulsions as a function of the composition of the ingredient used to stabilize the interface (Fig. 3B and C middle, Fig. 3 D). Since only two main crystalline structures (i.e. α 2L (46 Å) and α 3L (72 Å)) were identified by XRD, the interpretation of the DSC curves exhibiting at least four overlapped exotherms was complex. The first main exotherm was related to the formation of the α 2L (46 Å) crystals while the main second exotherm was related to the complex and then related to the α 3L (72 Å) crystals (Fig. 2, Fig. 3).

#### 3.2.2 Melting behaviour

**Fig. 4:**
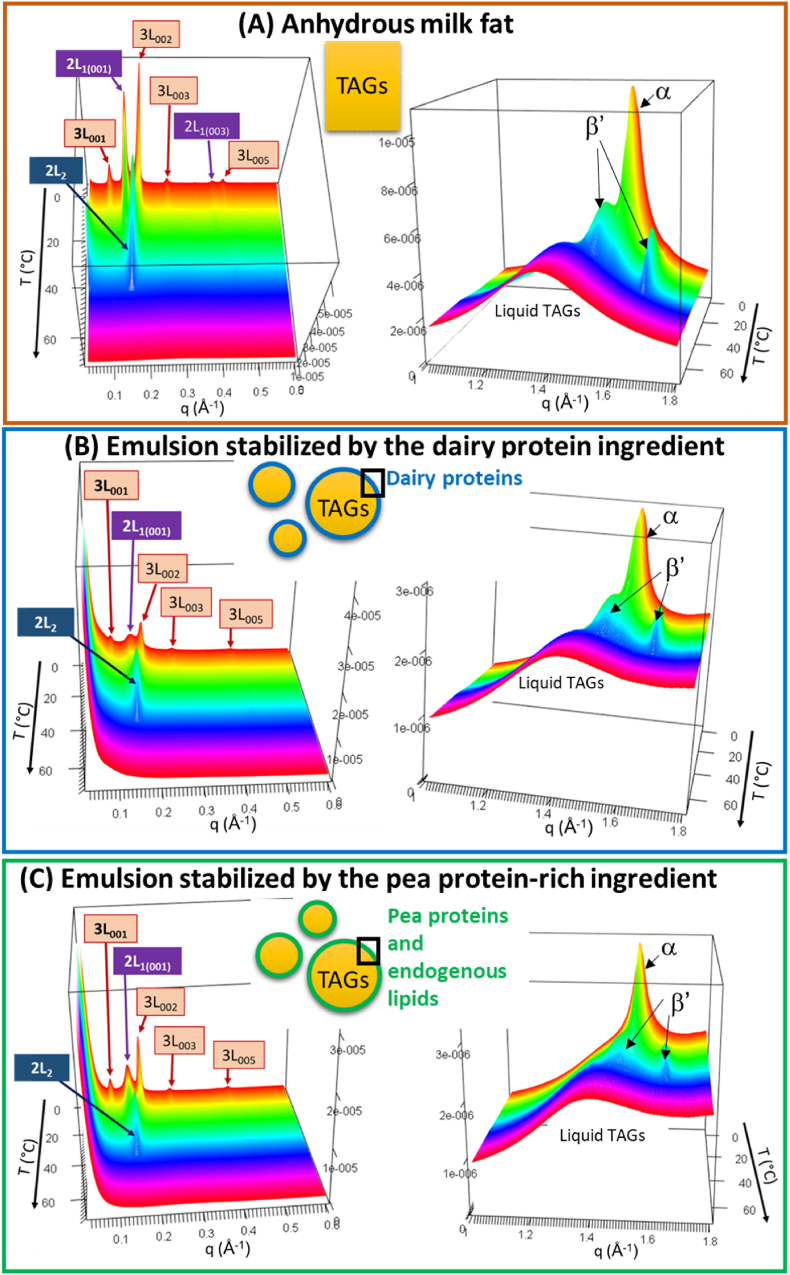
Three-dimensional plots of the synchrotron radiation X-ray diffraction (XRD) patterns recorded simultaneously at small (SAXS, left) and wide (WAXS, right) angles on heating at 3 °C/min of **(A)** anhydrous milk fat, **(B)** Oil-in-water emulsions stabilized by the dairy protein ingredient and **(C)** Oil-in-water emulsion stabilized by the pea protein-rich ingredient (isolate). The crystalline phases are identified and the reflexions are noted (Miller index). Abreviations: TAGs = triacylglycerols; 2L = double chain length; 3L = triple chain length; T = temperature; q = scattering vector.

After cooling at 3 °C/min from 65 °C to −5 °C, anhydrous milk fat and the O/W emulsions stabilized either by the dairy proteins or the pea protein-rich ingredient were heated at 3 °C/min from −5 °C to 60 °C. The DSC curves recorded on heating are presented Fig. 2 B. The 3D-representations of the synchrotron-radiation XRD patterns recorded simultaneously at small and wide angles as a function of time on heating between −5 °C and 60 °C at dT/dt = 3 °C/min of anhydrous milk TAGs and milk TAGs dispersed in the emulsions stabilized either by the dairy proteins or the pea protein-rich ingredient are presented Fig. 4.

The DSC curves recorded on heating of anhydrous milk TAGs and of the emulsions showed 5 temperature-delimited behaviours, noted from 1 to 5 in Fig. 2 B. The synchrotron-radiation XRD experiments performed as a function of temperature on heating (Fig. 4)**,** associated with previous reports (Lopez and Ollivon, 2009b), permitted an understanding of the structural reorganizations that successively occurred. The first endotherm observed below 10 °C (noted 1) corresponded to the melting of the α 3L crystals. Structural reorganizations exhibiting both endothermic and exothermic events occurred on heating (noted 2). The endotherm around 14 °C was attributed to the melting of the α 2L crystals. The exotherm recorded around 16.5–18 °C corresponded to the α to β′ polymorphic transition associated with structural reorganizations leading to the formation of new β′ 2L crystals noted 2L_2_ (Fig. 4). The endotherm recorded from 20 to 30 °C (noted 3) was attributed to the melting of TAGs and the last endotherm (noted 4) above 30 °C corresponded to the final melting of milk TAGs organised in β′ 2L_2_ crystals. Above the final melting point, the TAGs were fully liquid (noted 5, Fig. 2 B). Between 10 °C and 30 °C, the DSC curve recorded for the emulsion stabilized by the pea protein-rich ingredient showed a different behaviour of milk TAGs as compared to the other 2 systems. The separation between the two last endotherms recorded at 30 °C was attributed to the successive melting of 2 groups of milk TAGs involved in β’ 2L_2_ crystals. This different melting behaviour could have technological, functional and sensory implications that would deserve to be further studied.

Supercooling, characterized by the difference between the final temperature of melting and the initial temperature of crystallization (ΔT = T_offset_ – T_onset_) of milk TAGs, was observed in anhydrous state and in the emulsions. The ΔT values were as follows: ΔT = 18.0 °C for anhydrous milk TAGs, ΔT = 25.2 °C for the emulsion stabilized by the dairy protein ingredient and ΔT = 24.7 °C for the emulsion stabilized by the pea protein ingredient (Fig. 2 B). The supercooling means that the TAGs remained in their liquid state below their final temperature of melting where they could crystallize. This is in agreement with the literature (Lopez et al., 2001, 2002). The supercooling is explained by the thermodynamic metastability of the TAGs and by the mechanisms of nucleation (homogeneous or heterogeneous) and crystal growth occurring on cooling (Sato, 2001, 2018).

#### 3.2.3 Impact of the TAGs dispersion within lipid droplets and the key role played by components from the pea protein-rich ingredient on the crystallization and melting properties of milk TAGs

DSC and XRDT experiments showed that similar crystalline structures were formed on cooling of milk TAGs in anhydrous state or dispersed in emulsion lipid droplets: α 2L(46 Å) and then α 3L(72 Å) crystals. They were attributed to the successive crystallization of HCT and LCT milk TAGs. However, the dispersion of TAGs in emulsion lipid droplets induced a supercooling that has been recorded by DSC and XRDT and also induced changes in the MHPW of the (001) reflection XRD peaks. The MHPW of the first peak recorded on cooling and corresponding to the α 2L(46 Å) crystals was significantly smaller in anhydrous state than in emulsions (Fig. 3 D). The broadening of the XRD peak in the emulsions was attributed to small size and/or defaults in TAG crystals due to interfacial contraints in lipid droplets (Guinier, 1964).

In the emulsions stabilized by the pea protein-rich ingredient or the dairy proteins, differences in the liquid to solid α 2L(46 Å) TAG phase transition were recorded by DSC and XRDT. For the lipid droplets stabilized by the pea protein-rich ingredient, a first exotherm was recorded by DSC from 17 °C (Fig. 2 A) and attributed to crystallization of HCT milk TAGs in the α 2L(46 Å) structure (Fig. 3 B). The nucleation and growth rates of the α 2L(46 Å) crystals were faster in the lipid droplets stabilized by the pea protein-rich ingredient than in the lipid droplets stabilized by the dairy protein ingredient (Fig. 3B and C, middle). Also, the MHPW of the (001) reflection XRD peak related to the α 2L(46 Å) cystals was significantly smaller for the emulsion containing lipid droplets stabilized by the pea protein-rich ingredient than for the emulsion containing lipid droplets stabilized by the dairy proteins (Fig. 3 D). This could be related to a high level of organization of TAG molecules in the crystals induced by components present in the pea protein-rich ingredient. The results obtained by DSC and XRDT showed a specific behavior of milk TAGs in the emulsion stabilized by the pea protein-rich ingredient and suggested that the nucleation of TAGs was induced from the surface of the lipid droplets (surface-induced crystallization). Therefore, we deduced that the composition of this pea protein-rich ingredient and then that the molecules located at the TAG/water interface affected the liquid to solid α 2L(46 Å) TAGs phase transition. The concentration and the organization of the molecules located at TAG/water interface, i.e. proteins adsorbed and/or non-protein components such as pea-EL contained in the ingredient, could be involved in the mechanisms of nucleation of milk TAGs within the lipid droplets. Surface heterogeneous nucleation involved in the crystallization of TAGs within emulsion lipid droplets has been previously observed and attributed to amphiphilic components such as proteins or lipid components (e.g., monoacylglycerols, diacylglycerols, phospholipids) (Bunjes and Koch, 2005; Lopez and Ollivon, 2009a; Douaire et al., 2014).

We hypothesized that the composition of the pea protein-rich ingredient, either the pea proteins or the non-protein components such as the pea-EL, could be involved in the induction of milk TAGs crystallization within the lipid droplets.

### 3.3 Focus on the pea endogenous lipids (pea-EL) present in the protein-rich ingredient: composition, physical state as a function of temperature, and impact on the crystallization and melting properties of milk TAGs

In this part of the work, the lipid content of the pea protein isolate Nutralys® S85 Plus N was determined and the molecular species of the pea-EL were examined. Then, the physical state of the pea-EL was characterized as a function of temperature. Finally, the impact of the pea-EL on the crystallization and melting properties of milk TAGs in bulk and in emulsions was investigated.

#### 3.3.1 Composition of the pea-EL recovered from the pea protein isolate

**Fig. 5:**
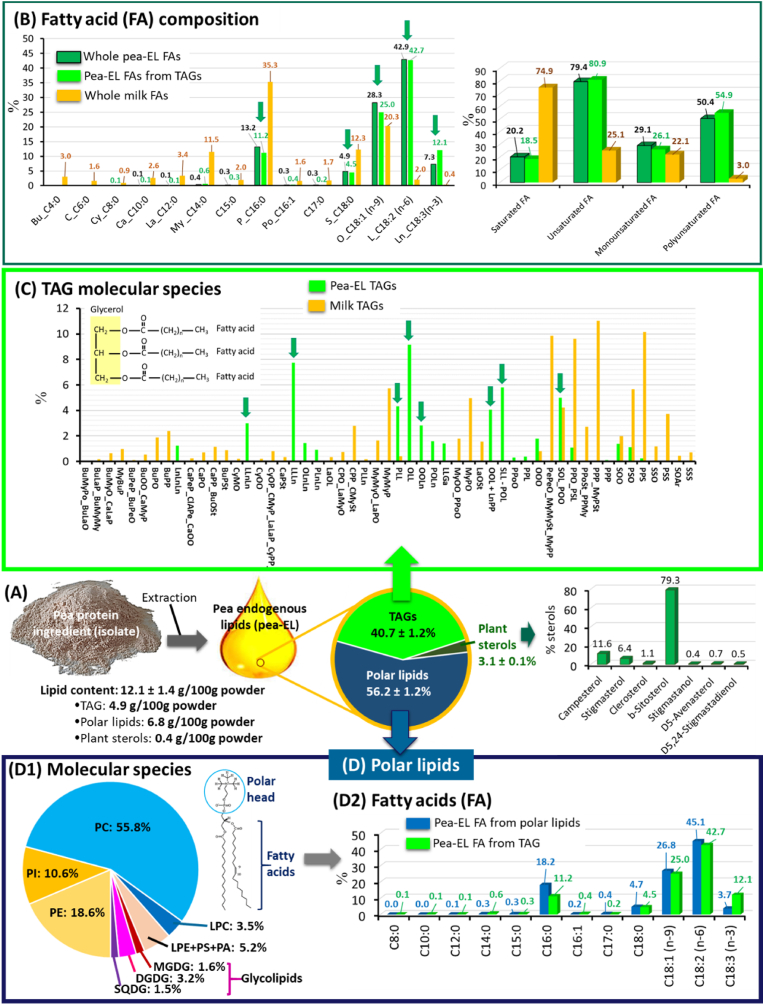
Lipid compositions of the pea protein isolate and milk fat: **(A)** total lipid content and main lipid species contained in the pea protein isolate called the pea endogenous lipids (pea-EL); **(B)** Fatty acid compositions **(C)** comparative analysis of the TAG molecular species in the pea-EL and milk fat; **(D)** polar lipids contained in the pea-EL, (D1) molecular species and (D2) fatty acid composition including a comparison with the fatty acid composition of pea-EL TAGs. Abreviations: TAGs = triacylglycerols; FAs: fatty acids; PC = phosphatidylcholine; PE = phosphatidylethanolamine; PI = phosphatidylinositol; PS = phosphatidylserine; LPC = lyso-phosphatidylcholine; LPE = lysophosphatidylethanolamine; PA = phosphatidic acid; MGDG = monogalactosyldiacylglycerol; DGDG = digalactosyldiacylglycerol; SQDG = sulfoquinovosyldiacylglycerol.

The pea protein isolate Nutralys® S85 Plus N contained 12.1 ± 1.4 %wt lipids in the powder (Fig. 5). This is in agreement with literature since authors reported that some pea protein isolates can have lipid contents of 10 %wt or more (Coxon and Wright, 1985). This high amount of endogenous lipids recovered in the pea protein-rich ingredient resulted from their concentration upon processing from peas (1–4 %wt lipids), as already reported (Coxon and Wright, 1985; Hall et al., 2017). The pea-EL were composed of 40.7 ± 1.2 %wt apolar neutral lipids that corresponded to TAG molecules (TAGs: 4.9 g/100g powder). The polar lipid fraction accounted for 56.2 ± 1.2 %wt of the total pea-EL (polar lipids: 6.8 g/100g powder). This relative amount of TAGs and polar lipids was in agreement with their relative proportion in the pea seeds (Hoover et al., 1988), which means that the pea protein isolate manufacturing process did not fractionate nor enrich specific species of pea lipids. This lipid composition of the pea protein isolate Nutralys® S85 Plus N is close to results recently reported for the Nutralys® S85F ingredient (Roquette, France) that is used for low protein content formulations (Keuleyan et al., 2023). Plant sterols accounted for 3.1 ± 0.2 %wt of the total pea-EL (plant sterols: 0.4 g/100g powder; unsaponifiable fraction), with β-sitosterol representing 79.3 % of the whole sterols, in agreement with the main amount of β-sitosterol in pea seeds (Miyazawa et al., 1974). The presence of plant sterols in pea protein-rich ingredients that resulted from their natural localization within pea lipid membranes has been poorly described (Miyazawa et al., 1974; Hoover et al., 1988). We are facing the increased incorporation of pea protein-rich ingredients in foods. In this context, the presence of plant sterols should be further considered particularly in infant formulas (Le Roux et al., 2020) and baby foods where it can compete with cholesterol (naturally provided by the milk fat globule membrane in human milk or added in infant milk formula) in infants and the potential long-term consequence (Plat et al., 2019).

The fatty acid compositions of the whole pea-EL and of the apolar TAG fraction are presented Fig. 5 B in comparison with the fatty acid composition of the milk TAGs. Five main fatty acids accounted for 95.5–96.5 % of the whole fatty acids present in the pea-EL, i.e., the unsaturated fatty acids C18:2 *n-*6 (main fatty acid), C18:1 *n-*9, and C18:3 *n-*3 and the saturated fatty acids C16:0 and C18:0. This is in agreement with the whole fatty acid composition of pea seeds (Coxon and Wright, 1985). The C18:2 *n-*6 (ω6)/C18:3 *n-*3 (ω3) ratio was 5.9 ± 0.1 in the whole pea-EL and 3.5 ± 0.1 in the pea TAGs. This high amount of unsaturated fatty acids in the pea-EL could explain the high susceptibility of pea protein-rich ingredients to oxidative rancidity leading to sensory defaults (Hoover et al., 1988). Compared to milk fat, the whole pea-EL contained a significantly lower amount of saturated fatty acids (20.2 vs. 74.9%), higher amount of unsaturated fatty acids (79.4 vs. 25.1%) with a higher amount of monounsaturated (29.1 vs. 22.1 %wt) and polyunsaturated (50.4 vs. 3.0 %wt) fatty acids (Fig. 5 B). The pea-EL and the milk fat therefore exhibited very different fatty acid compositions.

The molecular species of the TAGs recovered in the pea protein-rich ingredient (pea endogenous TAGs) are presented Fig. 5 C in comparison with milk TAGs. In the pea-EL, 8 TAG molecular species accounted for more than 2 %wt. In agreement with the fatty acid composition that contains high proportions of C18:1 *n-*9 (oleic acid, noted O), C18:2 *n-*6 (linoleic acid, noted L) and C18:3 *n-*3 (linolenic acid, noted Ln), the pea endogenous TAGs were mainly triunsaturated (25.4 % of the TAGs): OLL (9.1 %), LLLn (7.8 %), LLnLn (3 %), OOLn (2.8 %), OLnLn (1.45 %), LnLnLn (1.25 %). Other pea TAGs contained 1 saturated fatty acid and 2 unsaturated fatty acids: SLL-POL (5.8 %), SOL-POO (5 %), PLL (4.3 %). Milk TAGs contained a wide diversity of TAG molecular species, with a high proportion of TAGs containing 3 long-chain saturated fatty acids such as MyPP, MyMyP, PPP, PPS, PSS, and 2 long-chain saturated fatty acids combined with 1 unsaturated fatty acid such as PPO, PSO and MyPO, as previously described (Lopez et al., 2006). The comparative analysis of the TAG molecular species revealed that the pea protein-rich ingredient contains low melting temperature TAGs composed mainly of unsaturated fatty acids while the dairy protein ingredient contains high melting temperature TAGs rich in saturated long-chain fatty acids (Fig. 5 C).

Among pea-EL present in the pea protein isolate Nutralys® S85 Plus N, the high amount of endogenous polar lipids (56.2 ± 1.2 %wt of pea-EL) could play functional roles mainly at the TAG/water interface in emulsions. Indeed, polar lipids are amphiphilic molecules with a polar head group and hydrophobic acyl chains that can locate at the surface of lipid droplets in food emulsions and act as natural emulsififiers. The composition of the pea polar lipid fraction was therefore deeper characterized. The polar lipid fraction of the pea-EL contained a range of molecular species (Fig. 5 D), including the phospholipids (phosphatidylcholine: PC, phosphatidylethanolamine: PE, the anionic phosphatidylinositol: PI and phosphatidylserine: PS), lysophospholipids (LPC and LPE) and glycolipids (sugar headgroup) (Fig. 5 D1). The main phospholipid was PC that accounted for about 56 %wt of the polar lipids. This polar lipid composition of pea-EL was in agreement with previous reports (Hoover et al., 1988; Keuleyan et al., 2023). The fatty acid composition of the pea-EL polar lipid fraction was mainly represented by 5 fatty acids, as for the whole pea-EL and pea TAGs. The content in the saturated long chain C16:0 was significantly higher in the pea polar lipid fraction than in the pea TAG fraction: 18.2 vs 11.2 %wt (Fig. 5 D2).

The pea-EL contained a wide range of lipid molecular species with different structures and physicochemical properties (e.g., molecular shape, polarity, charge of the polar head, degree of unsaturation and length of the fatty acyl chains), leading to potential different implications in the functional properties of the pea protein-rich ingredient. Particularly, the saturated long chain fatty acid C16:0 exhibits a high temperature phase transition and we hypothesized that it could be involved in functional properties of the pea protein-rich ingredient as regard to TAG crystallization.

#### 3.3.2 Thermal properties of pea-EL

**Fig. 6:**
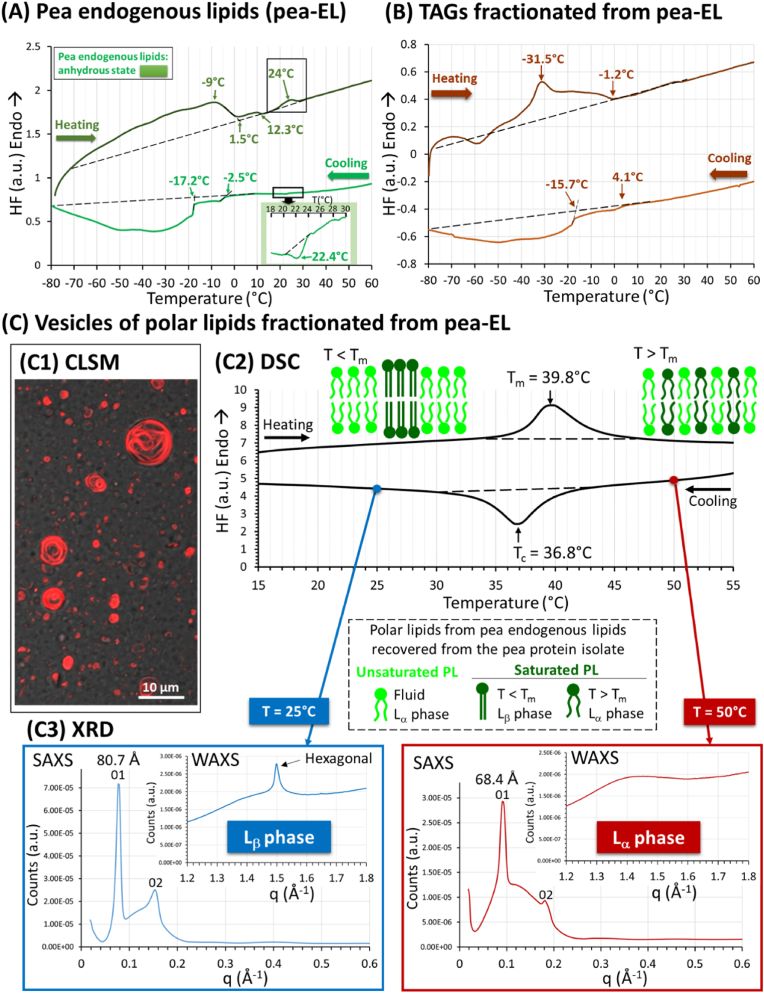
Physical properties of pea endogenous lipids as a function of temperature. **(A)** Differential scanning calorimetry (DSC) curves recorded on cooling and subsequent heating at 3 °C/min of pea endogenous lipids (pea-EL); **(B)** DSC curves recorded on cooling and subsequent heating at 3 °C/min of triacylglycerols (TAGs) fractionated from the pea endogenous lipids. **(C)** Thermal and structural properties of the polar lipids fractionated from pea endogenous lipids: **(C1)** Confocal laser scanning microscopy image combined with transmitted light showing the vesicles of pea endogenous polar lipids (red: fluorescent dye Nile red used to label the bilayers of pea endogenous phospholipids); **(C2)** DSC curves recorded on cooling and subsequent heating at 3 °C/min fully hydrated vesicles of pea endogenous polar lipids. Schematic representation of the assemblies of polar lipids as a function of temperature. **(C3)** Synchrotron radiation X-ray diffraction (XRD) patterns recorded simultaneously at small (SAXS) and wide (WAXS) angles at 25 °C and 50 °C. The phases are identified and the reflexions are noted (Miller index). Abreviations: T = temperature; T_m_ = temperature of melting; Tc = temperature of crystallization; PL; polar lipids; q = scattering vector. (For interpretation of the references to colour in this figure legend, the reader is referred to the Web version of this article.)

Fig. 6 shows the DSC curves recorded on cooling and subsequent heating of the anhydrous pea-EL. On cooling, the DSC curve showed a small exotherm at 22.4 °C and then two overlapped and broad exotherms spanning from −2.5 °C to −80 °C. Three main endotherms were recorded on heating: a broad endotherm spanning from about −70 °C to 1.5 °C, a second endotherm spanning to 12.3 °C, and a third endotherm spanning from 19 to 28 °C with a maximum at 24 °C. The main broad exotherm and endotherm were interpreted as the crystallization and melting of TAG molecules from the pea-EL. The low crystallization and melting temperatures were in agreement with the high proportions of polyunsaturated fatty acids and triunsaturated TAGs present in the pea-EL (Fig. 5B and C). The exotherm recorded at 22.4 °C was attributed to a liquid to solid phase transition occurring on cooling of anhydrous pea-EL. The low enthalpy corresponding to this transition (0.03 J/g; 0.2 % of the whole enthalpy of crystallization) may correspond to quantitatively minor lipid components present in the pea-EL. The endotherm recorded at 24 °C could correspond to the melting of these components present in pea-EL (0.33 J/g; 1.3 % of the whole enthalpy of melting). We hypothesized that this thermal transition could be due to the presence of polar lipids containing saturated long-chain fatty acids (C16:0).

Fig. 6 B shows the DSC curves recorded on cooling and subsequent heating of the TAGs fractionated from pea-EL. A broad exotherm was recorded below 4 °C. The thermal behaviour recorded on heating was more complex with the succession of an endotherm below −65 °C, an exotherm that could correspond to a phase transition, and a broad endotherm spanning until the final melting of pea TAGs at −1.2 °C. The thermal behaviour of pea TAGs exhibiting changes in their physical state below 0 °C was in agreement with the high proportions of polyunsaturated fatty acids and triunsaturated TAGs in the pea-EL (Fig. 5B and C).

Fig. 6 C shows the DSC curves recorded on cooling and subsequent heating of the polar lipids fractionated from pea-EL as well as the identification of the structures formed at 25 °C and 50 °C performed by XRD. The pea polar lipids recovered from the whole pea-EL were hydrated at 60 °C and vortexed to form multilamellar vesicles (Fig. 6 C1), and to investigate their thermal and structural properties as a function of temperature. The vesicles of pea endogenous polar lipids exhibited a phase transition as a function of temperature. The DSC curve recorded on cooling showed an exotherm with T_c_ = 36.8 °C, and an endotherm with T_m_ = 39.8 °C was recorded on heating (Fig. 6 C2). XRD recorded simultaneously at both small and wide angles permitted the identification of the structural organization of the pea polar lipids above and below the range of temperatures corresponding to the phase transition (Fig. 6 C3). At 50 °C, i.e. for T > T_c_ and T_m_, the pea polar lipids were organized as lamellar structures with a thickness of 68.4 Å (q = 0.09185 Å^−1^) with a fluid liquid-crystalline organization characterized by a bump of diffusion at wide angles. This XRD signature corresponded to the L_α_ organization of the pea polar lipids. At 25 °C, i.e. for T < T_c_ and T_m_, the pea polar lipids were organized as lamellar structures with a thickness of 80.7 Å (q = 0.07786 Å^−1^). The single peak of diffraction recorded at wide angles was attributed to the formation of a hexagonal packing of the acyl chains. This XRD signature corresponded to the ordered gel (L_β_) organization of the pea polar lipids below their temperature of melting. The difference Δd = 12.3 Å recorded between lamellar organisation in the L_β_ and L_α_ phases is in agreement with the elongation of the acyl chains in an ordered packing.

The L_α_ to L_β_ phase transition occurring on cooling of the pea polar lipid fraction could be attributed to saturated phospholipids such as DPPC that exhibits two phase transitions in the range 37–41 °C (Grabielle-Madelmont and Perron, 1983; Murthy et al., 2015). Indeed, The phospholipid palmitoyl(16:0)-oleoyl(18:1)-PC exhibits a phase transition at T_m_ = −2 °C and the fully unsaturated PC dioleoyl(18:1)-PC (DOPC) exhibits a very low temperature of L_β_ to L_α_ (gel to fluid) phase transition at T_m_ = −20 °C (Et-Thakafy et al., 2017). The L_α_ to L_β_ phase transition occurring on cooling of the pea polar lipid fraction could also involve the monogalactosyldiacylglycerol (MGDG) that is rich in C16:0 (27.5% according to (Hoover et al., 1988)) and exhibits high phase transition temperature in mixture with saturated PC such as DPPC due to strong H-bonding interactions occurring between the sugar headgroup of MGDG and the phosphate, carbonyl and choline groups of PC that could contribute to the phase behavior (Popova and Hincha, 2011).

These DSC and XRD experiments revealed that pea polar lipids contain high T_m_ species that are in an ordered L_β_ state above the initial temperature of crystallization of milk TAGs (Fig. 2).

#### 3.3.3 Impact of pea-EL on the crystallization and melting properties of milk TAGs in anhydrous state and in O/W emulsions

##### 3.3.3.1 Impact of pea-EL on milk TAGs crystallization in anhydrous state

**Fig. 7:**
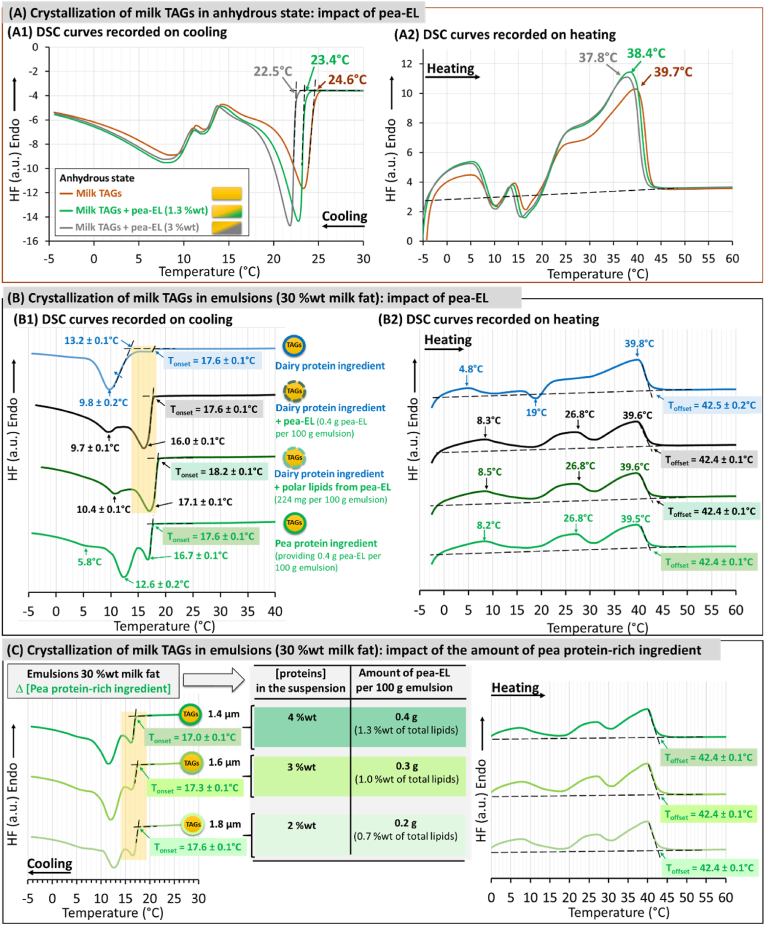
Impact of pea endogenous lipids (pea-EL) on the crystallization and melting properties of milk fat in anhydrous state and in emulsions. **(A)** Differential scanning calorimetry (DSC) curves recorded on cooling (A1) and subsequent heating (A2) at 3 °C/min of milk TAGs alone or with the addition of various amounts of pea-EL (1.3 %wt and 3 %wt). **(B)** DSC curves recorded on cooling (B1) and on subsequent heating (B2) at 3 °C/min of emulsions stabilized by the dairy protein ingredient (top), the dairy protein ingredient with the addition of pea-EL or polar lipids from pea-EL (middle) and the pea protein ingredient (bottom). **(C)** DSC curves recorded on cooling (left) and on subsequent heating (right) at 3 °C/min of emulsions prepared with various amounts of the pea protein-rich ingredient leading to various amounts of pea proteins (from 4 to 2 %wt) and pea endogenous lipids as indicated in the figure; the mean diameters of the lipid droplets determined by laser light scattering are also indicated in the figure. Abreviations: TAGs = triacylglycerols; T_onset_ = initial temperature of crystallization; T_offset_ = final temperature of melting.

Fig. 7 A shows the DSC curves recorded on cooling and subsequent heating of milk TAGs, and milk TAGs supplemented with pea-EL at 2 different amounts (1.3 %wt and 3 %wt of total lipids). The amount of 1.3 %wt pea-EL corresponds to the amount of pea-EL naturally provided by the pea protein isolate in the O/W emulsions. The addition of pea-EL to milk TAGs did not change the DSC profiles recorded on cooling (i.e. the number of exotherms and their shape), but induced a decrease in the initial temperature of crystallization (T_onset_): T_onset_ = 24.6 °C for milk TAGs, 23.4 °C for milk TAGs + pea-EL (1.3 %wt), and 22.5 °C for milk TAG + pea-EL (3 %wt). We deduced that this decrease in the initial temperature of crystallization in presence of pea-EL was due to the solubility between milk TAGs and the liquid unsaturated fatty acid rich TAGs contained in pea-EL (Fig. 5B and C) that delayed the formation of the first TAGs crystals on cooling. On heating, the addition of pea-EL to milk TAGs did not change the DSC profiles but induced a decrease in the temperature of the last endotherm and then on the final melting temperature of the TAGs. From these results, we deduced that upon homogenization at 50 °C (Fig. 1), melted milk TAGs and liquid TAGs from pea-EL can mix together in the lipid droplets and affect both the whole TAG composition and the crystallization properties of milk TAGs by introducing low melting point pea TAGs.

##### 3.3.3.2 Impact of pea-EL on milk TAGs crystallization in O/W emulsion: key interfacial role played by the polar lipid fraction of pea-EL

In order to decipher the role played by pea-EL from the pea protein-rich ingredient on the induction of milk TAGs crystallization, we prepared O/W emulsions containing lipid droplets coated by the dairy proteins alone or with the addition of pea-EL or polar lipids fractionated from pea-EL (noted PL_pea-EL).

The O/W emulsions prepared with a homogenization pressure of 4 MPa exhibited monomodal size distributions with different mean diameters of the lipid droplets depending on the composition (i.e., dairy protein ingredient, dairy protein ingredient + pea-EL, dairy protein ingredient + PL_pea-EL pea protein-rich ingredient). The mean diameters were 2.24 ± 0.01 μm for dairy protein ingredient, 1.27 ± 0.06 μm for dairy protein ingredient + pea-EL, 1.25 ± 0.04 μm for dairy protein ingredient + PL_pea-EL and 1.40 ± 0.06 μm for pea protein ingredient (results not shown). These results showed that the presence of pea-EL or PL_pea EL in the emulsions containing dairy proteins decreased the mean diameter of the lipid droplets (Δd = −0.97 μm for P = 4 MPa). This was interpreted as a consequence as the surface-active properties of the phospholipids present in the pea-EL. These results demonstrated that pea-EL that are naturally present in the pea protein-rich ingredient (12.1 %wt of the dry matter, Fig. 5) play a functional role during the preparation of O/W emulsions and stabilization of the lipid droplets.

In a second step of the experiments, we adapted the homogenization pressure to prepare emulsion lipid droplets with similar size distributions (2.2 ± 0.1 μm) in order to investigate the impact of pea-EL on the crystallization properties of milk TAGs in O/W emulsions. Fig. 7 B shows the DSC curves recorded on cooling and subsequent heating of the emulsion lipid droplets stabilized by the dairy protein ingredient, the dairy protein ingredient supplemented with the pea-EL or PL_pea-EL, and the pea protein-rich ingredient. The addition of pea-EL or PL_pea-EL in the formulation of the emulsions stabilized by the dairy protein ingredient induced, on cooling from the melt, the formation of a high temperature exotherm (Fig. 7 B1). This exotherm was interpreted as the crystallization of milk TAGs within the lipid droplets. This exotherm was similar to the exotherm recorded on cooling of the emulsions prepared with the pea protein-rich ingredient that naturally contained 12.1 %wt of pea-EL. These experiments revealed the surface-active role played by pea-EL, and more specifically by the polar lipid fraction of pea-EL, on the induction of milk TAGs crystallization in O/W emulsion. The initial temperature of crystallization was the same in the O/W emulsions whatever the composition of the interface, T_onset_ = 17.6 ± 0.1 °C, except for the emulsion containing PL_pea-EL T_onset_ = 18.2 ± 0.1 °C. This high T_onset_ was attributed (i) to the absence of liquid TAGs from pea-EL that did not induce a delay in the initial crystallization of milk TAGs (Fig. 7 A), and (ii) to the specific action of polar lipids in the ordered phase (Fig. 6), located at the surface of the lipid droplets. Considering the delay in anhydrous milk TAGs crystallization observed in presence of pea-EL (liquid TAGs), we could consider that the initial temperature of crystallization recorded for the O/W emulsion (Fig. 2, Fig. 7) results from both the presence of liquid TAGs from pea-EL (delay in the crystallization of milk TAGs) and the induction of crystallization due to polar lipids from pea-EL located at the TAG/water interface.

Fig. 7**B2** shows the DSC curves recorded on heating of the O/W emulsions. The final melting point of TAGs was T_offset_ = 42.4 ± 0.2 °C for the 4 kinds of emulsions. The DSC profiles were different as a function of the surface composition of the lipid droplets. On heating of the O/W emulsion formulated with the dairy protein ingredient, the DSC curve revealed a first endotherm with a maximum at 4.8 °C, then an endotherm and an exotherm with a minimum at 19 °C, and finally a broad endotherm spanning from 20 °C to the final melting temperature T_offset_ = 42.5 ± 0.2 °C. The addition of pea-EL or PL_pea-EL in the formulation of the O/W emulsions affected the melting profile that ressembled that of the O/W emulsions prepared with the pea protein-rich ingredient that naturally contains pea-EL including the polar lipid fraction (56.2 %wt of pea-EL, Fig. 5). In the O/W emulsions containing pea-EL, 3 main endotherms were successively recorded on heating with maxima at 8.3 °C, 26.8 °C and 39.6 °C.

These experiments demonstrated, for the first time to the author's knowledge, the key role of pea-EL on the induction of milk TAGs crystallization in O/W emulsions, and revealed that at least part of the pea-EL were located at the TAG/water interface.

Fig. 7 C shows the DSC curves recorded on cooling and subsequent heating at 3 °C/min of the O/W emulsions prepared with various amounts of pea protein-rich ingredient in order to modulate the amount of proteins and pea-EL in the formulations. The decrease in the amounts of pea protein-rich ingredient induced the decrease in the protein content from 4 %wt to 2 %wt and the amount of pea-EL decreased from 0.4 to 0.2 g per 100 g emulsion. Homogenized at 4 MPa, the O/W emulsions exhibited an increase in the mean diameter of the lipid droplets as a function of the decrease in protein and pea-EL contents, which revealed the impact of the formulation (amount of ingredient to stabilize the TAG/water interface). On cooling from the melt, the 3 emulsions exhibited a first sharp exotherm and then two other exotherms (Fig. 7 C, left). We deduced that the surface-induced crystallization of TAGs operated even at 0.2 g pea-EL per 100 g emulsion (0.66 %wt lipids provided by pea-EL). Changes in the initial temperature of milk TAGs crystallization were related to differences in the mean diameters of the lipid droplets, as previously reported (Lopez et al., 2002). The DSC curves recorded on heating of the emulsions did not show any difference due to the formulation (Fig. 7 C, right).

### 3.4 General discussion: from pea protein-rich ingredients to the surface-active properties of pea endogenous lipids on the crystallization properties of milk fat

This study revealed, for the first time to the authors’ knowledge, a key interfacial and functional role played by pea-EL in O/W emulsions. The high amount of pea-EL in the pea protein isolate, i.e. 12.1 ± 1.4 g per 100 g powder (Fig. 5), resulted from the protein isolate manufacturing process. The pea-EL assemblies recovered in a wet process could be polar lipid coated TAG droplets, vesicles and plant membranes (Fig. 1). The pea-EL contained 56.2 ± 1.2% polar lipids among which phospholipids such as the zwitterionic PC (about 56 %wt of pea endogenous polar lipids, Fig. 5D), and the anionic PI and PS that are charged molecules sensitive to pH. We therefore emit the hypothesis that the acidic pH used to precipitate the pea proteins for the preparation of the protein isolate also induced simultaneously the precipitation of pea lipid assemblies at their isoelectric point. This raises the question of the impact of protein ingredient manufacturing processes on the composition of the plant-based ingredients including the amount of non-protein components such as endogenous lipids and on the functional consequences in food applications.

**Fig. 8:**
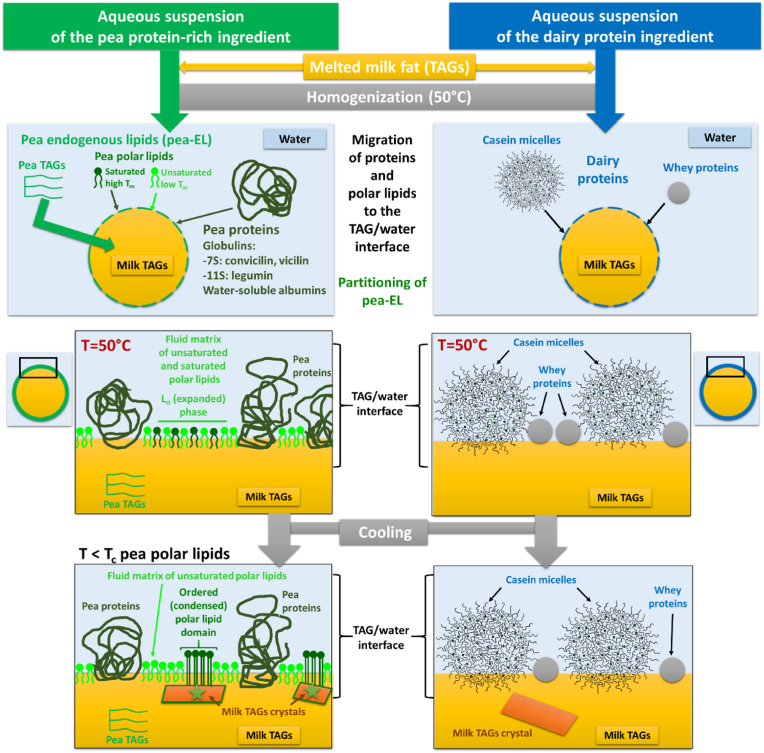
Pea protein-rich ingredient versus dairy proteins: schematic representation of the mechanisms involved in the shaping of the architecture of the TAG/water interfaces formed upon processing involving homogenization and subsequent decrease in temperature. At 50 °C, the partitioning of pea endogenous lipids in their liquid state occurred: pea triacylglycerols (TAGs) migrated in the core of the lipid droplets where they mixed with milk TAGs while pea polar lipids migrated together with pea proteins to the TAG/water interface. The decrease in temperature induced phase transition of the saturated pea polar lipids with changes in their physical state leading to the formation of condensed domains involved in templating and in the surface-induced crystallization of milk TAGs. Dairy proteins adsorbed at the TAG/water interface. Not to scale.

Fig. 8 shows a schematic representation of the mechanisms involved in the shaping of the TAG/water interface in the O/W emulsions stabilized either by the pea protein-rich ingredient or by dairy proteins that had consequences on the crystallization properties of milk TAGs.

Upon homogenization performed at 50 °C, that is a key step in the formation of emulsion lipid droplets, and to subsequent cooling of the O/W emulsions, several mechanisms occurred that deserve to be further discussed. The large insoluble powder grain particles present in the aqueous suspension of pea proteins (Fig. 1 B) were disrupted under shear and the pea-EL assemblies were released in the aqueous phase as observed by CLSM (results not shown). The pea proteins in their aggregated and/or native colloidal states (both globulins and albumins) migrated towards and adsorbed to the TAG/water interface to minimize the interfacial tension between the liquid TAGs and the water phases. Then, the pea proteins probably reoriented at the TAG/water interface through structural rearrangements so their hydrophobic moieties were located towards the TAG phase and the hydrophilic residues are facing the water phase (Drusch et al., 2021). The O/W emulsions prepared in this study were physically stable during 15 days, which demonstrated the efficacity of the protein-rich ingredients to stabilize the lipid droplets.

Upon the homogenization process performed at 50 °C in presence of melted milk TAGs, pea-EL partitioned as a function of their polarity, i.e. apolar TAGs or polar lipids, and exhibited different spatial localizations in the O/W emulsion (Fig. 8). The pea apolar TAGs composed of unsaturated fatty acids (Fig. 5B and C) migrated towards the TAG phase and mixed with milk TAGs in the liquid state as deciphered by DSC (Fig. 7). The interior of the lipid droplets was therefore composed of a mixture of TAGs from milk and pea. The impact of the unsaturated fatty acid rich pea TAGs on the oxidative degradation of the dietary fat used to prepare the O/W emulsion, from milk as in this study or from other sources (e.g., PUFA rich oils such as rapeseed oil) depending on the formulation of the emulsion, remains to be elucidated.

This study provided evidence, by the combination of DSC and XRDT techniques (Fig. 2, Fig. 3, Fig. 7), for the localization of pea endogenous polar lipids at the surface of the lipid droplets where they played a key interfacial role by inducing milk TAGs crystallization. The preferential localization of pea polar lipids at the surface of the lipid droplets is due to their amphiphilic structure adapted to the TAG/water interface. Moreover, this study revealed that the pea endogenous polar lipids do not have to be considered as a single category. Indeed, the acyl chains of the pea polar lipids (saturated or unsaturated) govern their thermodynamic properties, their physical state as a function of temperature and their functional role at the TAG/water interface. Low melting temperature (low T_m_) pea polar lipids, rich in unsaturated acyl chains (C18:2 *n-*6, C18:3 *n-*3; Fig. 5) were located at the TAG/water interface where they formed a fluid matrix whatever the temperature. Among the pea polar lipids adsorbed at the TAG/water interface, those containing saturated long acyl chains (C16:0, i.e. DPPC and MGDG) exhibited high melting temperature (high T_m_) and changed their physical state on cooling (Fig. 6 C). In the ordered (condensed) phase, the molecular packing of these high T_m_ saturated pea polar lipids locally increased at the O/W interface leading to their lateral phase separation and to the formation of ordered lipid domains (Fig. 8). Plant sterols, that are present in the pea protein isolate (Fig. 5), could be solubilized among the polar lipids at the TAG/water interface, as in natural lipid membranes. The ordered lipid domains may have served as interfacial templates for surface heterogeneous nucleation and crystal growth of high crystallization temperature (HCT) milk TAGs (formation of α 2L 46 Å crystals; Fig. 3) that are rich in C16:0 and C18:0 fatty acids (Fig. 5 A). Our results and interpretations are in agreement with previous studies reporting that in O/W emulsions the arrangement of emulsifier molecules adsorbed at the O/W interface can create a matrix which templates the interfacial heterogeneous nucleation of the TAGs present in the oil phase (Douaire et al., 2014). Moreover, authors reported that for such a templating effect to occur, the hydrophobic tail of the emulsifier and the TAGs should exhibit some level of structural similarity, that is the case between pea saturated polar lipids and HCT milk TAGs. The templating effect requires hydrophobic molecular interactions between the acyl chains of the pea saturated polar lipids and the milk TAGs within the lipid droplets. Molecular interaction can occur when chains match in length and structure, thus exhibiting enough structural similarities with the oil to induce nucleation (Awad and Sato, 2001; Lopez and Ollivon, 2009a; Douaire et al., 2014). Surface heterogeneous nucleation involved in the crystallization of TAGs within emulsion lipid droplets has been previously observed and attributed to amphiphilic components such as proteins or lipid components (e.g., monoacylglycerols, diacylglycerols, phospholipids) (Bunjes and Koch, 2005; Lopez and Ollivon, 2009a; Douaire et al., 2014). Saturated phospholipids such as DPPC have been shown to interact with crystallizing TAGs (Smith, 2000; Bunjes and Koch, 2005; Lopez and Ollivon, 2009a). At the O/W interface, the carbon tails of the pea endogenous saturated polar lipids were orientated normal to the interface and as a consequence crystal growth may have resulted in the formation of crystal platelets parallel to this interface (Fig. 8), as previously reported (Arima et al., 2009).

Changes in the crystallization characteristics of milk TAGs induced by the pea-EL could have consequences on the physical stability of the lipid droplets (e.g., during churning of cream to prepare spreads), on the rheological properties of TAG crystal networks, and on the texture of the final product. These aspects require further investigations.

The complex architecture of the TAG/water interface formed with the pea protein-rich ingredient could have other functional implications. The TAG/water interface exhibited heterogeneities in terms of composition (protein-rich areas *versus* lipid-rich areas), topography and physical properties (rigid lipid domains, fluid matrix of polar lipids, soft colloidal particles of proteins) (Fig. 8). The lateral phase separation between saturated high T_m_ polar lipids in the ordered (condensed) phase that form lipid domains and unsaturated low T_m_ polar lipids in the fluid (expanded) phase has been reported for natural lipid assemblies in biological membranes (Lopez et al., 2010; Lopez, 2020b) and in model systems (Murthy et al., 2016). Atomic force microscopy experiments reported the higher height and rigidity of the ordered lipid domains compared to the fluid matrix (Murthy et al., 2016; Et-Thakafy et al., 2017, 2019). The impact of temperature on the physical state of polar lipids and then on their lateral phase separation has been previously reported (Murthy et al., 2016; Et-Thakafy et al., 2019). The TAG/water interface should therefore be considered as a dynamic system that evolves as a function of temperature. Moreover, the physical properties and topography of lipid assemblies in membranes or hemi-membranes can govern the insertion of proteins. The preferential localization of proteins including digestive enzymes (e.g., lipases) has been reported to be in the fluid matrix of polar lipids or at the boundary of ordered lipid domains (Bourlieu et al., 2016; Obeid et al., 2019). This raises questions about the role played by pea endogenous polar lipids located at the TAG/water interface on the mechanisms of digestion of food emulsions at physiological temperature in the gastrointestinal tract.

## 4 Conclusion

The growing demand for sustainable plant-based protein sources has stimulated interest in new ingredients. The use of pea protein-rich ingredients as alternatives to dairy proteins questioned the potential impact of their chemical complexity, and particularly the presence of endogenous lipids, on the crystallization properties of dairy emulsions. In this study, we showed that the complexity of pea protein-rich ingredients such as isolates composed of 12 %wt endogenous lipids in the powder (56.2 %wt polar lipids, 40.7 %wt triacylglycerols, 3.1 %wt plant sterols) impacted the interfacial and functional properties of the dairy O/W emulsions. Pea endogenous lipids (pea-EL) partitioned upon homogenization at 50 °C as a function of their polarity. The pea apolar lipids (triacylglycerols, TAGs) mixed with milk TAGs in their melted state while the pea polar lipids located at the TAG/water interface. Both pea proteins (convicilin, legumin, vicilin, albumins) and endogenous polar lipids (phospholipids and glycolipids) from the pea protein isolate were present at the TAG/water interface due to their amphiphilic and surface-active properties. Pea polar lipids played different roles as a function of the saturation of their fatty acid chains and physical state. This study revealed that high T_m_ saturated pea polar lipids that changed their physical state on cooling played a key interfacial role in the heterogeneous surface-induced crystallization of milk TAGs within emulsion lipid droplets. These findings highlight the importance of the characterization of protein ingredients including plant-based ingredients to better understand and control their functional properties in food formulations.

## CRediT authorship contribution statement

**Christelle Lopez:** Conceptualization, Supervision, Investigation, Formal analysis, Validation, Writing – original draft, Writing – review & editing. **Magalie Weber:** Conceptualization, Investigation, Formal analysis, Validation, Writing – original draft, Writing – review & editing. **Hanitra Rabesona:** Conceptualization, Investigation, Formal analysis, Validation, Writing – original draft, Writing – review & editing. **Javier Pérez:** Conceptualization, Investigation, Formal analysis, Validation, Writing – original draft, Writing – review & editing. **Franck Artzner:** Conceptualization, Investigation, Formal analysis, Validation, Writing – original draft, Writing – review & editing. **Thomas Bizien:** Conceptualization, Investigation, Formal analysis, Validation, Writing – original draft, Writing – review & editing.

## Declaration of competing interest

The authors declare that they have no known competing financial interests or personal relationships that could have appeared to influence the work reported in this paper.

## Data Availability

Data will be made available on request.
